# *Pantoea ananatis* Genetic Diversity Analysis Reveals Limited Genomic Diversity as Well as Accessory Genes Correlated with Onion Pathogenicity

**DOI:** 10.3389/fmicb.2018.00184

**Published:** 2018-02-13

**Authors:** Shaun P. Stice, Spencer D. Stumpf, Ron D. Gitaitis, Brian H. Kvitko, Bhabesh Dutta

**Affiliations:** ^1^Department of Plant Pathology, University of Georgia, Athens, GA, United States; ^2^Department of Plant Pathology, University of Georgia, Tifton, GA, United States; ^3^The Plant Center, University of Georgia, Athens, GA, United States

**Keywords:** *Pantoea ananatis*, pangenome, onion, CWDE, MLSA, bacterial secretion systems

## Abstract

*Pantoea ananatis* is a member of the family Enterobacteriaceae and an enigmatic plant pathogen with a broad host range. Although *P. ananatis* strains can be aggressive on onion causing foliar necrosis and onion center rot, previous genomic analysis has shown that *P. ananatis* lacks the primary virulence secretion systems associated with other plant pathogens. We assessed a collection of fifty *P. ananatis* strains collected from Georgia over three decades to determine genetic factors that correlated with onion pathogenic potential. Previous genetic analysis studies have compared strains isolated from different hosts with varying diseases potential and isolation sources. Strains varied greatly in their pathogenic potential and aggressiveness on different cultivated *Allium* species like onion, leek, shallot, and chive. Using multi-locus sequence analysis (MLSA) and repetitive extragenic palindrome repeat (rep)-PCR techniques, we did not observe any correlation between onion pathogenic potential and genetic diversity among strains. Whole genome sequencing and pan-genomic analysis of a sub-set of 10 strains aided in the identification of a novel series of genetic regions, likely plasmid borne, and correlating with onion pathogenicity observed on single contigs of the genetic assemblies. We named these loci Onion Virulence Regions (OVR) A-D. The OVR loci contain genes involved in redox regulation as well as pectate lyase and rhamnogalacturonase genes. Previous studies have not identified distinct genetic loci or plasmids correlating with onion foliar pathogenicity or pathogenicity on a single host pathosystem. The lack of focus on a single host system for this phytopathgenic disease necessitates the pan-genomic analysis performed in this study.

## Introduction

*Pantoea ananatis* is a member of the Enterobacteriaceae family and is a ubiquitous phytopathogenic bacterium capable of infecting diverse monocotyledonous hosts including maize, rice and pineapple as well as the woody host, Eucalyptus (Serrano, 1928; Tabei et al., 1988; Paccola-Meirelles et al., 2001; Coutinho et al., 2002; Coutinho and Venter, 2009). *P. ananatis* strains have also been recovered from opportunistic infections in humans (De Baere et al., 2004). In the United States, *P. ananatis* is of primary concern as a causative agent of center rot of onion (*Allium cepa* L.). The first outbreak of center rot of onion in the United States occurred in Georgia in 1997 and since then it has been identified in different onion growing regions (Gitaitis and Gay, 1997; Schwartz and Otto, 2000; Carr et al., 2010). *P. ananatis* outbreaks are a recurring challenge for onion production and outbreaks resulting in considerable crop loss have been documented (Walcott et al., 2002). *P. ananatis* infection can be transmitted via seed but it is also a common epiphyte on weeds and is known to be transmitted transiently between plants by two different species of thrips (Gitaitis et al., 2002; Walcott et al., 2002; Dutta et al., 2014). Infection of onions is facilitated through wounds or thrips damage. *P. ananatis* strains are known to express the ice-nucleation active (InaZ) cell surface protein which efficiently nucleates ice crystals and is associated with increased incidence of frost damage (Lindow, 1983; Abe et al., 1989). Upon leaf invasion, *P. ananatis* reaches high levels in leaf blades and causes foliar necrosis. The pathogen can also progress from leaf blade to the corresponding onion bulb scale where, alongside colonization by secondary pathogens, it causes center rot symptoms in central scales (Carr et al., 2013).

Management and accurate diagnosis of *P. ananatis* has been complicated due to the phenotypic variation among different strains in onion pathogenicity. *P. ananatis* strains collected from non-onion sources (thrips, weeds) may either be highly aggressive on onions or completely onion non-pathogenic. Currently no strain features have been described that can predict onion pathogenic potential.

*Pantoea ananatis* is an enigma among bacterial phytopathogens. Whole genome sequencing has shown that phytopathogenic *P. ananatis* strains lack Type II, Type III, and Type IV virulence-associated secretion systems as well as known phytotoxin synthesis genes (De Maayer et al., 2014; Sheibani-Tezerji et al., 2015; Weller-Stuart et al., 2017). In the absence of these pathogenicity factors, secretion systems used to deliver Cell Wall Degrading Enzymes (CWDEs) outside of the bacterium (i.e., T2SS), or virulence effector proteins directly into the plant cells (i.e., T3SS/T4SS); however, it remains an open question as to what host interaction mediated pathogenicity factors are associated with *P. ananatis* infection of onion. Progressing leaf necrosis has been shown to be dependent on pathogen quorum sensing and swimming motility (Morohoshi et al., 2007). In addition, the Type VI Secretion System (T6SS), most commonly associated with inter-bacterial competition, was shown to play a role in foliar necrosis symptoms but with little effect on pathogen populations (Morohoshi et al., 2007; Shyntum et al., 2015; Weller-Stuart et al., 2017).

In this study we assessed a collection of fifty Georgia *P. ananatis* strains collected over three decades (1997–2015) from diverse sources to determine what genetic factors correlated with onion pathogenic potential. Using multi-locus sequence analysis (MLSA) and repetitive extragenic palindrome (rep)-PCR techniques, we did not observe strong correlations between onion pathogenic potential and genetic diversity among strains. We found that strains varied greatly in their pathogenic potential and aggressiveness on different cultivated *Allium* species like onion, shallot (*Allium cepa* var. *aggregatum*), chives (*Allium schoenoprasum*), and leeks (*Allium porrum*). Several strains that were non-pathogenic on onion displayed significant virulence on leek. Pan-genome analysis derived from whole genome sequencing of a sub-set of six onion pathogenic and four onion non-pathogenic Georgia strains identified four loci associated only with the six onion pathogenic strains. These four loci were present on a single contig. We named these loci as the Onion Virulence Regions A through D (OVR-A-D). The OVR loci contains pectate lyase and rhamnogalacturonase genes as well as genes that are involved in redox regulation.

## Materials and methods

### Bacterial strains, identification, and culture preparation

Fifty *P. ananatis* strains used in this study were isolated from onion and other sources including weeds and thrips throughout the state of Georgia (Table 1). These strains were stored in sterile 15% aqueous glycerol solution at −80°C. The strains which were isolated from either symptomatic onion foliage, bulb, or from contaminated onion seeds were designated as “PNA” in our culture collection. Strains isolated from asymptomatic weeds or from the gut of thrips (*Thrips tabaci* and *Frankliniella fusca*) were designated as “PANS.” The source, year of isolation, and county of origin in Georgia for these strains are shown in Table 1. These strains were identified as *P. ananatis* by their morphological and physiological characteristics such as Gram-negative, facultative anaerobic, positive for indole production, negative for nitrate reductase and phenylalanine deaminase and by PCR amplification of a 398 base pair (bp) fragment using *P. ananatis* species-specific primers (Gitaitis et al., 2002).

**Table 1:** Isolates of *Pantoea ananatis* and their associated phenotypic characteristics.

**Strain Name^a^**	**Host**	**Place of isolation in Georgia (county)**	**Ice nucleation^b^**	**Copper tolerance^c^**	**Strain aggressiveness^d^**			
					**Onion**	**Chive**	**Leek**	**Shallot**
PANS 99-1	*Richardia scabra* L.	Tift	−	−	+++	−	+	+
**PANS 99-3**	*Richardia scabra* L.	Tift	−	−	+++	−	−	+
PANS 99-11	*Digitaria sanguinalis*	Tift	+	−	+++	−	−	−
PANS 99-27	*Desmodium tortuosum*	Toombs	+	−	+++	−	−	+
PANS 99-29	*Digitaria sanguinalis*	Tift	+	−	+++	−	−	+
**PANS 01-2**	*Thrips tabaci*^T^	Tift	+	−	+++	−	+	+
**PNA 97-1**	*Allium cepa* L.	Toombs	+	−	+++	−	+	+
PNA 98-8	*Allium cepa* L.	Toombs	+	−	+++	−	++	+
PNA 99-3	*Allium cepa* L.	Tift	+	−	+++	−	++	+
PNA 200-11	*Allum cepa* L. seed	Tift	+	−	+++	+	−	+
PNA 02-18	*Allium cepa* L.	Tattnall	+	−	+++	−	−	++
**PNA 06-1**	*Allium cepa* L.	Wayne	+	−	+++	−	−	+
PNA 07-1	*Allium cepa* L.	Tattnall	+	−	+++	−	++	−
PNA 08-1	*Allium cepa* L.	Tattnall	+	−	+++	−	++	+
**PNA 15-1**	*Allium cepa* L.	Tattnall	−	−	+++	−	+	+
PANS 99-22	*Digitaria sanguinalis*	Tift	−	−	++	−	++	−
PANS 01-5	*Thrips tabaci*^T^	Tift	+	−	++	−	++	+
PANS 01-6	*Thrips tabaci*^T^	Tift	+	−	++	−	+++	++
PNA 07-5	*Allium cepa* L.	Wayne	−	−	++	−	−	++
PNA 13-1	*Allium cepa* L.	Toombs	−	−	++	−	+	++
PNA 14-1	*Allium cepa* L.	Toombs	−	−	++	−	−	−
PNA 99-14	*Allium cepa* L.	Toombs	+	−	+	−	−	++
PNA 14-4	*Allium cepa* L.	Toombs	−	−	+	−	−	−
PANS 99-24	*Vigna unguiculata*	Toombs	−	−	+	−	+	−
**PNA 200-3**	*Allium cepa* L. seed	Tift	−	−	+	ND	ND	ND
PANS 99-33	*Richardia scabra* L.	Coffee	−	−	Y	ND	ND	ND
PANS 99-33	*Richardia scabra* L.	Coffee	−	−	Y	ND	ND	ND
PANS 02-5	*Frankliniella fusca*^T^	Tift	+	−	Y	ND	ND	ND
PNA 97-11	*Allium cepa* L.	Toombs	+	−	Y	ND	ND	ND
PNA 98-2	*Allium cepa* L.	Tift	+	−	Y	ND	ND	ND
PNA 98-11	*Allium cepa* L.	Tattnall	+	−	Y	ND	ND	ND
PNA 99-2	*Allium cepa* L.	Tattnall	+	−	Y	ND	ND	ND
PNA 99-8	*Allium cepa* L.	Wheeler	+	−	Y	ND	ND	ND
**PANS 99-23**	*Cyperus esculentus*	Toombs	+	−	−	−	+++	−
PANS 99-26	*Chamaesyce hyssopifolia*	Toombs	+	−	−	−	++	−
PANS 99-6	*Glandularia bipinnatidifida*	Tift	−	−	−	−	++	−
PANS 200-1	*Amaranthus viridis*	Toombs	+	−	−	−	−	+
**PNA 99-7**	*Allium cepa* L.	Tattnall	−	−	N	ND	ND	ND
PANS 99-5	*Glandularia bipinnatidifida*	Tift	−	−	N	ND	ND	ND
PANS 99-32	*Richardia scabra* L.	Toombs	+	−	−	−	−	−
**PANS 99-36**	*Richardia scabra* L.	Terrell	+	−	−	−	−	−
PANS 02-1	*Frankliniella fusca*^T^	Tift	−	−	−	−	−	−
**PANS 04-2**	*Frankliniella fusca*^T^	Tift	−	−	−	ND	ND	ND
PNA 99-9	*Allium cepa* L.	Tattnall	+	−	N	ND	ND	ND
PNA 200-7	*Allium cepa* L. seed	Tift	+	−	N	ND	ND	ND
PNA 200-8	*Allium cepa* L. seed	Tift	−	−	N	ND	ND	ND
PNA 200-12	*Allium cepa* L. seed	Tift	+	−	N	ND	ND	ND
PNA 05-1	*Allium cepa* L.	Tattnall	+	−	N	ND	ND	ND
PNA 07-10	*Allium cepa* L.	Toombs	+	−	N	ND	ND	ND
PNA 11-1	*Allium cepa* L.	Toombs	−	−	N	ND	ND	ND

a *First digits of the strain designation indicate year of isolation, e.g., 97 = 1997, 200 = 2000*.

b *Ice-nucleation test at −5°C*.

c Growth on nutrient agar amended with 200 ppm CuSO_4_ *5H_2_O and colony growth were observed after 48–72 h after incubation.

d *Strain aggressiveness on the foliage of onion, chive, leek, and shallot 5 dpi. Strains lacking pathogenicity are designated by a “−,“ weakly aggressive strains on a host are represented by a “+,” moderately aggressive “++,” and highly aggressive “+++” Y/N denote previously recorded as pathogenic or non-pathogenic on onion*.

T *Species of thrips, a vector insect associated with the P. ananatis center rot pathosystem*.

*Bold names indicate whole genome sequencing (WGS) strains*.

Inoculum was prepared by transferring single colonies of each bacterial strain from 48-h old cultures on nutrient agar (NA) medium to nutrient broth or lysogeny broth (LB). The broth was shaken overnight on a rotary shaker (Inova; New Brunswick Scientific, Edison, NJ) at 150 rpm. After a 12-h of incubation, 10 ml of each bacterial suspension were centrifuged at 6,000 × *g* (Allegra 25R, Beckman Coulter, Fullerton, CA) for 5 min. The supernatant was discarded and the pellet was re-suspended in 0.1 M phosphate-buffered saline (PBS). Later, inoculum was adjusted using a spectrophotometer (Spectronic 20; Bausch and Lomb, Rochester, NY) to an optical density of 0.3 at 600 nm [≈1 × 10^8^ colony forming unit (CFU)/ml]. The bacterial suspension was serially diluted in PBS to obtain the desired concentration according to each experiment. PNA 97-1R is a spontaneous rifampicin (Rf) resistant clone of PNA 97-1 recovered after plating a dense suspension of PNA 97-1 onto an LB Rf 60 plate.

### Multi-locus sequence analysis (MLSA) of *P. ananatis* strains

Total microbial genomic DNA was extracted from a 3 ml volume of overnight cultures grown at 28°C in nutrient broth using an UltraClean Microbial DNA Kit (MO BIO, Carlsbad, CA) according to the manufacturer's instructions. Extracted genomic DNA was quantified on a NanoDrop 1000 spectrophotometer (Thermo Scientific, Wilmington, U.S.A.) and 50 ng of genomic DNA were used for each PCR reaction. Six primer pairs of housekeeping genes, *fusA, gyrB, leuS, pyrG, rpl*, and *rpoB*, were chosen based on a previous MLSA scheme (Salerno et al., 2007; Deletoile et al., 2009). The concatenation of these six genes represented a total of 2,961 bp. Primer sequences and PCR cycles are outlined in Supplementary Table 3. Each PCR reaction was performed in a 50 μl reaction consisted of 10 μl of 5x PCR buffer, 0.2 mM of dNTPs, 0.2 μM of each primer, 1.25 U HotStart Taq Polymerase (Qiagen, Valencia, Ca) and 50 ng/μl of template DNA. PCR products were subjected to gel electrophoresis in Tris-acetate-EDTA buffer on a 1.5% agarose gel at 100 V for 45 min. Reactions resulting in positive amplifications and expected fragment size were purified with a DNA Clean and Concentrator Kit (Zymo Research Inc., Irvine, Ca), according to the manufacturer's instructions. Fragment sequencing was conducted by Eurofins, (Louisville, KY).

### Phylogenetic analysis of sequencing data

Sequences were manually trimmed and verified based on peak quality of forward and reverse chromatograms in Geneious 8.1.8 (Biomatters, Auckland, New Zealand) and were aligned using MUSCLE (http://www.ebi.ac.uk/Tools/msa/muscle/). The nucleotide sites presenting alignment gaps were excluded from analysis and were then concatenated. Consensus sequences were used to generate a maximum-likelihood (ML) tree based on the Tamura-Nei Model of MEGA 6.06 with bootstrapping repetitions of 1,000. Sequences from *P. agglomerans* 97-1 (accession no. FN434113.1) from GenBank were used as outgroups. MLST sequences generated from this study were deposited in GenBank under accession numbers MF925240–MF925289, MF939901–MF939950, MF939951–MF940000, MF940001–MF940050, MF940051–MF940100, and MF964629–MF964678.

### Repetitive extragenic palindrome (rep)-PCR amplification and analysis

Genomic DNA from strains were isolated as described above and subjected to rep-PCR using REP1R-I (III ICG ICG ICA TCI GGC) and REP 2-I (ICG ICT TAT CIG GCC TAC) primers (Versalovic et al., 1991). The amplification was carried out in a total of 25 μl reactions containing 50 ng of DNA, 10 mM Tris-HCl, 50 mM KCl, 1.5 mM MgCl_2_, 200 μM dNTPs, 2.5 U pureTaq DNA polymerase, and 0.5 μM of each primer pair. Amplifications were done using the following conditions: an initial denaturation at 95° C for 4 min, 45 cycles consisting of 94°C for 1 min, 45°C for 1 min, and 65°C for 8 min, followed by cooling for 4 min. Samples were separated by gel electrophoresis in Tris-acetate-EDTA buffer on a 1.5% agarose gel for 2 h at 100 V stained with GelGreen. DNA fingerprinting profile for each *P. ananatis* strain were compared using Dice's (1945) coefficient of analysis with the aid of BioNumerics software package (Applied Math, Kortrijk, Belgium), and the unweighted pairwise group method with arithmetic mean algorithm was used to generate a dendrogram indicating strain relatedness.

### Aggressiveness of *P. ananatis* strains on onion, chive, leek, and shallot

The aggressiveness of 33 selected strains were tested on 6-week old shallots (cv. “Camelot F1”), chives (cv. “Dolores”), leeks (cv. “King Richard”), and onion (cv. “Century”) under greenhouse conditions. Strains from 11 clades (*n* = 23) and from unresolved portion (*n* = 10) of the MLSA phylogenetic tree were selected for this assay. These strains represented 66% of the total *P. ananatis* strains from 11 clades and unresolved portion. Seedlings of each plant species were established in 10 × 8 cm (diameter × height) plastic pots (Hummert International, Earth City, MO) containing commercial potting mix. The seedlings were kept in greenhouse conditions and maintained at 25–28°C and 80–90% RH with a 12L:12D photoperiod. Seedlings were fertilized with osmocote smart-release plant food (14-14-14) (Scotts Miracle-Gro Company, Marysville, OH). Inoculum preparation and standardization were conducted as described above. Seedlings were inoculated by cutting the central leaf 1 cm from the apex with a sterile pair of scissors. Using a micropipette, a 10 μl drop of bacterial suspensions containing 1 × 10^8^ CFU/mL (≈1 × 10^6^ CFU) were placed at the cut end of the seedlings. Seedlings mechanically inoculated with PBS as described above were used as negative controls. Four replications per strain per host were used in a single experiment and this experiment was repeated one more time. The experiment was arranged in a randomized complete block design. Seedlings were checked visually for symptom development every day until 5 days after inoculation (DAI). At 5 DAI, lesion length for each seedling was measured and mean lesion length for each strain on each plant species were compared using Fisher's protected least significant difference test at *P* < 0.05 level.

To confirm that symptoms observed were caused by *P. ananatis*, bacteria from symptomatic tissue (*n* = 5 symptomatic plants per host) were isolated from a region adjoining the necrotic and healthy tissue and streaked onto Tryptic soy broth agar (TSBA) plates and incubated for 48-h at 28°C. After 48-h of incubation, yellow-pigmented colonies were isolated and tested for *P. ananatis* using physiological tests (as previously described) and species-specific TaqMan based polymerase-chain reaction (PCR) assays (Walcott et al., 2002). Briefly, presumptive *P. ananatis* colonies were picked using a sterile inoculation loop and suspended separately in a 2 ml micro-centrifuge tubes containing 25 μl of sterile deionized water. The bacterial suspension was heated (Modular Dry Block Heaters, Cole Parmer, IL) for 3 min at 95°C. A suspension (5 μl) was amplified in 20 μl of PCR master-mix containing 10 mM Tris-HCl (pH 9.0), 50 mM KCl, 0.1% Triton X-100, 1.5 mM MgCl_2_, and 0.2 mM of each nucleotide (dATP, dCTP, dGTP, and dTTP), 25 μM each of primer PanITS1 (5′-GTCTGATAGAAAGATAAAGAC-3′) and EC5 (5′- CGGTGGATGCCCTGGCA-3′) and 10 μM of TaqMan probe 6-FAM TAGCGGTTAGGACTCCGCCCTTTCA-BHQ. The PCR reaction was conducted in a Cepheid Smart Cycler (Sunnyvale, CA) using the following thermal profile: denaturation at 95°C for 180 s, 35 cycles each of denaturation at 95°C for 15 s, and annealing at 60°C for 40 s. Samples with cycle threshold (Ct) values <35 were considered positive for *P. ananatis*.

### Red onion scale assay

Consumer produce red onions (*Allium cepa*. L., Red Onion Delana Farms, CA) were purchased, sliced to remove any diseased tissue, cut to approximately 3 cm wide scales, sterilized in a 3% bleach solution for 1 min and promptly removed and rinsed in dH_2_O. Scales were placed in a potting tray (27.0 × 52.0 cm) containing two layers of paper towels pre-moistened with 90 ml of distilled water. Individual onion scales were wounded on the inner surface with a sterile pipette tip, and 10 μl of a prepared bacterial suspension (previously described) (10^6^ CFU/ml) was inoculated into the wound. The tray was covered with a plastic humidity dome. The onion scales were incubated at room temperature for 72 h in the dark. The size of lesion necrotic zone and pigment-clearing zone was recorded to assess pathogenicity. Pathogenic strains that cleared the red anthocyanin pigment and caused maceration were labeled pathogenic and those that did not were labeled non-pathogenic. Six replicates were carried out for each strain. Sterile deionized water was used as negative control.

### Ice nucleation test

To test the ice nucleating ability of *P. ananatis* strains, five separate drops of 10 μl aliquots of bacterial suspension (>1 × 10^9^ CFU/ml) were pipetted onto a sterile weigh boat in an ice-ethanol water bath adjusted to −5°C. Sterile water was used as a negative control and PNA 97-1, an ice-nucleating strain, was used as a positive control. Samples that froze concurrently with the positive control were considered ice nucleation positive and samples that remained in liquid form or with delayed ice-nucleation activity were considered ice nucleation negative. Fifty *P. ananatis* strains were used and the experiment was repeated twice.

### Copper tolerance

Strains were grown and re-suspended to an optical density of 0.3 at 600 nm, as previously described, and suspensions were serially diluted to a concentration of 10^6^ CFU/ml. Aliquots of 50 μl were spread onto nutrient agar (NA) and NA amended with 200 ppm CuSO_4_^*^5H_2_O. Plates were incubated at 28C for 48–72 h and observed for bacterial growth.

### High quality draft genome of PNA 97-1R

Genomic DNA from the prototype strain PNA 97-1R was obtained from overnight cultures started from single colonies using the Gentra Puregene Yeast/Bact kit (Qiagen Inc), quantified using biospectrometer (Eppendorf), and visually assessed for quality on a 0.75% agarose gel. DNA quality, sequencing and assembly were carried out at the University of Delaware DNA Sequencing & Genotyping Center (Newark, DE). DNA was assessed for quality metrics via Fragment Analyzer (Advanced Analytical Technologies). Sequence reads were generated by a Pacific Biosciences RSII Single-Molecule DNA Sequencer following SMRT Bell library preparation. Basic *de-novo* assembly was carried out using PacBio HGAP assembler (v3) with seed reads 17 kilo base (kb) and above. Contigs were polished using Quiver and annotation was performed via Rapid Annotation using Subsystem Technology (RAST v2.0) or Prokaryote Genome Annotation Pipeline (PGAP v4.2.) (Zhao et al., 2012; Overbeek et al., 2014).

### Draft genomes of 10 diverse strains

Genomic DNA from 10 diverse strains (PNA 97-1R, PNA 06-01, PNA 200-3, PNA 15-1, PNA 99-7, PANS 04-02, PANS 01-02, PANS 99-23, PANS 99-36, and PANS 99-3) was obtained from overnight cultures started from single colonies using the Gentra Puregene Yeast/Bact Kit (Qiagen Inc.), quantified using Biospectrometer (Eppendorf), and visually assessed as above on a 0.75% agarose gel. Sequencing, assembly, annotation, and variant calling were carried out at MicrobesNG (Birmingham, U.K.). Sequence reads were generated by an Illumina HiSeq using 250-bp paired-end libraries, and assembled using SPAdes (version 3.10.1). Reads were mapped to the reference genome PNA 97-1R and back to contigs using BWA-MEM to ensure quality and 30X coverage. Annotation was performed via Prokka or RAST (Overbeek et al., 2014; Seemann, 2014).

### Pan-genomic analysis

The Prokka annotated fragmented *de novo* assemblies for the ten draft genomes for strains (PNA 97-1R, PNA 06-01, PNA 200-3, PNA 15-1, PNA 99-7, PANS 04-02, PANS 01-02, PANS99-23, PANS 99-36, and PANS 99-3) were loaded in GFF3 format to the ROARY (v3.6.1) program using default parameters to calculate the pan-genome of the 10 strains (Page et al., 2015). ROARY was run on a windows computer through an emulated Linux machine as described by the authors (https://sanger-pathogens.github.io/Roary/). The resulting newick tree files were edited using Geneious (v10.1.3). The “gene_presence_absence.csv” output file was analyzed using Microsoft excel to identify gene that are present in the pathogenic strains and absent in the non-pathogenic strains (Supplementary File 1).

The average nucleotide identity of the strains (ANI) was calculated using the online ANI calculator server with default parameters to compare WGS strains to the genomic type strain LMG 20103 (http://enve-omics.ce.gatech.edu/ani/). Additional Pan-genomic analysis was conducted using RAST BLASTP pairwise alignment with PNA-97-1 listed as reference genome. The computationally intensive Pan Genomic Analysis Pipeline (PGAP) was run on the UGA Sapelo computer cluster (Zhao et al., 2012). The resulting “5.Orthologs_Cluster_Function” file was opened with Microsoft excel and genes clustering in pathogenic strains and non-pathogenic strains were cross referenced with the results from ROARY analysis. The resulting putative virulence genes were then identified in the contigs of the draft genomes using Geneious. The contigs containing putative virulence associated genes were extracted and aligned using the Geneious Mauve plugin (v2.3.1) (Darling et al., 2004). Putative secretion target signals were assessed by running genes through the Signal P (v4.1) Server and TMHMM Server (v2.0) for prediction of transmembrane helices in proteins (Darling et al., 2004; Nielsen, 2017). Genomes were also investigated for secondary metabolite non-ribosomal peptide synthetase (NRPS) biosynthesis gene clusters using the antiSMASH bacterial server. Whole genome MLST (wgMSLT) was conducted using PGAdb-builder (Liu et al., 2016). The 95% occurrence allele scheme was selected to generate a wgMLST tree with *P. agglomerans* PC10 and *P. stewartii* subsp. *indologens* LMG 2632 selected as outgroups (Accession NZ_LIME00000000.1 and JPKO00000000.1). Genomic islands were identified using the IslandPath DIMOB function on IslandViewer4 server (Bertelli et al., 2017). Partial and complete phage regions were identified in the genome assemblies of the WGS strains using Phaster. Circular genome comparisons of WGS query strains and the OVR A-D locci to the reference strain PNA 97-1R were conducted using BRIG (GPLv3) (Alikhan et al., 2011).

### Nucleotide sequence accession numbers

The Whole Genome Shotgun project of *P. ananatis* strains (PNA 97-1R, PNA 06-1, PNA 200-3, PNA 15-1, PNA 99-7, PANS 04-2, PANS 01-2, PANS 99-23, PANS 99-36, and PANS 99-3) have been deposited at DDBJ/ENA/Genbank under the accessions (CP020943-CP020945), NMZY00000000, NMZX00000000, NMZZ00000000, NMZW00000000, NMZV00000000, NMZU00000000, NMZS00000000, NMZT00000000, and NMZR00000000, respectively. In addition, all bio-projects and accession numbers related to this sequencing project are linked under the NCBI umbrella project PRJNA400632.

## Results

### Multi-locus sequence analysis

**Figure 1:**
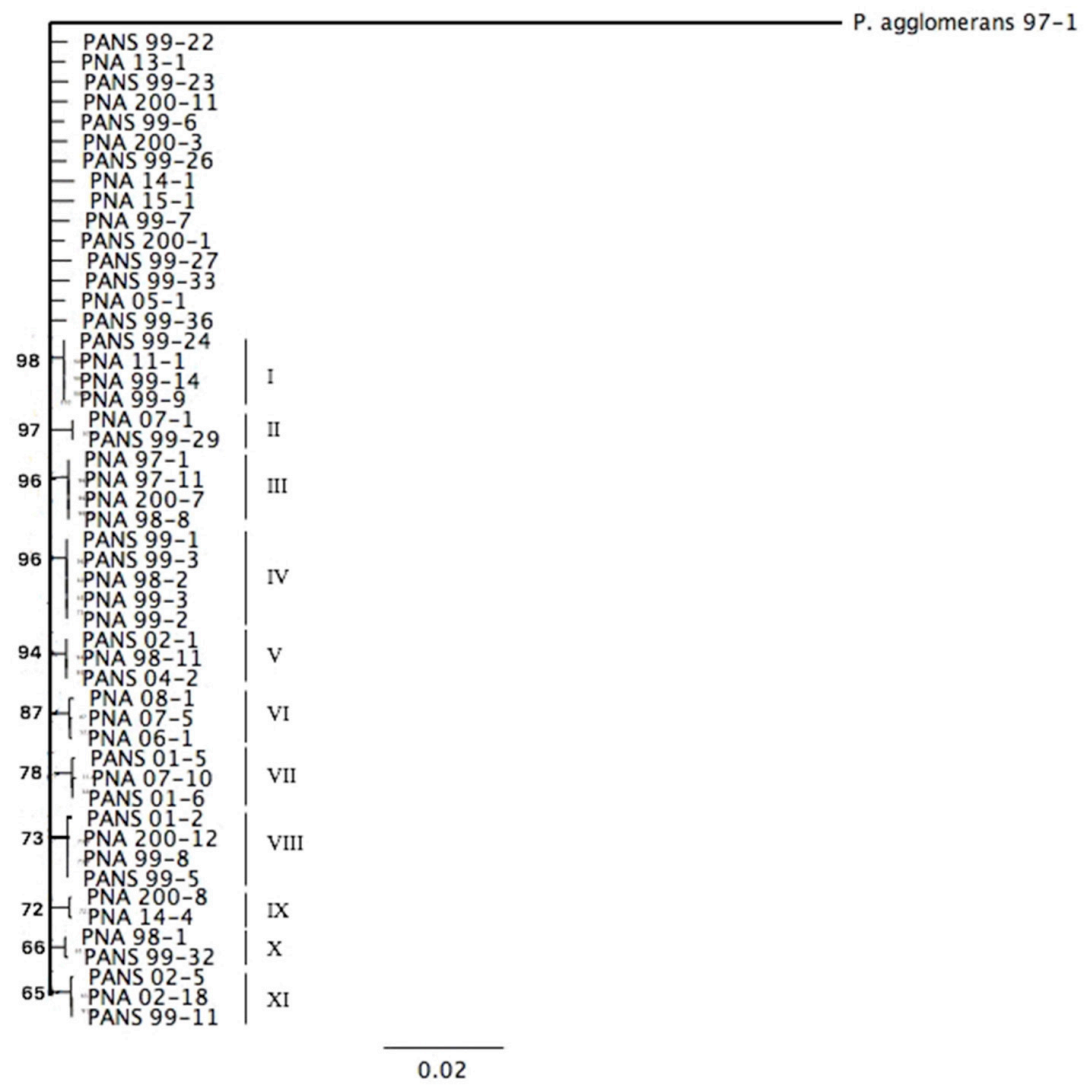
Maximum-likelihood tree based on the Tamura-Nei Model of *Pantoea ananatis* strains derived from concatenation of six housekeeping genes (*fusA, gyrB, leuS, pyrG, rplB, rpoB*). Bootstrap values are based on 1,000 repetitions and values >60% are shown in the figure.

Six housekeeping genes (*fusA, gyrB, leuS, pyrG, rpl*, and *rpoB*) from fifty *P. ananatis* strains were sequenced and a concatenated tree was developed. Sequence identity amongst the strains was >99% indicating low diversity. Overall, 11 small clades consisting of *P. ananatis* strains (*n* = 35) with bootstrap values >67 were observed (Figure 1). Among 11 clades, three clades contained strains, which were strictly isolated from onion or onion seed (designated PNA) (Figure 1). In addition, there were eight clades that contained strains isolated from diverse sources including onion or onion seed or weeds or thrips. None of the strains isolated from sources other than onion or onion seed (designated PANS) were exclusively resolved in a separate clade. Fifteen strains remain unresolved (Figure 1). There was no trend among strains with respect to their year of isolation, geographic location, source of isolation or ice-nucleation phenotype.

### Repetitive extragenic palindrome (rep)-PCR

A dendrogram was constructed from the rep-PCR profiles of fifty *P. ananatis* strains. Strains that showed relatedness more than 60% were clustered into four separate clades containing PNA and PANS strains. Fingerprint patterns were not correlated with isolation source or phenotype. Clade I strains (*n* = 24) were 80% similar while strains in Clade II (*n* = 11) had similarity of 86% (Supplementary Figure 1). Five strains displayed identical fingerprint profile within Clade III and were unique from the strains in the clades I–IV (Supplementary Figure 1). Clade IV comprised of ten strains that showed fingerprint profiles different from other clades and displayed 61% genetic similarity (Supplementary Figure 1).

### Pathogenicity and aggressiveness of *P. ananatis* strains on onion, chive, leek, and shallot

Thirty-three strains pathogenic or non-pathogenic on onion based on MLSA findings were selected for pathogenicity and aggressiveness study on onion, chive, leek, and shallot under controlled greenhouse conditions. PBS control plants displayed no foliar symptoms in any test. On onion, 100% of the previously determined pathogenic strains displayed typical foliar necrosis symptoms. However, there were significant differences among strains with respect to the aggressiveness (*P* < 0.0001; Table 1). Fifteen strains representing a mix of PNA and PANS strains were highly aggressive on onion with lesion length >7.8 cm after 5-days of inoculation under greenhouse conditions. Moderate levels of aggressiveness were displayed by ten strains also representing a mix of PNA and PANS strains. All eight previously determined onion non-pathogenic PANS strains were again determined to be non-pathogenic on onions.

On chive, all strains except PNA 200-11 were non-pathogenic. The PNA 200-11 strain was weakly aggressive on chive with lesion length of 0.92 cm after 5-days of inoculation. Out of 25 onion pathogenic strains, 19 strains were also pathogenic on shallot and they varied significantly in aggressiveness (*P* = 0.0007). These strains were weakly to moderately aggressive and they produced lesions of >0.6 cm after 5 days of inoculation. Six strains, which were pathogenic on onion, were non-pathogenic on shallot; however, one onion nonpathogenic strain, PANS 200-1 was found to be mildly aggressive on shallot. On leek, of the 25 onion pathogenic strains, 12 strains were also pathogenic and again, they varied significantly in aggressiveness (*P* < 0.0001). These strains were either weakly or moderately aggressive on leek with lesion length >0.8 cm after 5-days of inoculation. Thirteen onion pathogenic strains were non-pathogenic on leek. However, four onion non-pathogenic strains were weakly to moderately aggressive on leek. All symptomatic samples (*n* = 5 per host type) assayed were determined as positive for *P. ananatis* by a real-time PCR assay.

### Ice nucleation and copper tolerance

Ice nucleating ability was observed in 66% (33/50) of the strains tested. Of the 33 strains that were positive for ice nucleation, 18 were isolated from onion, three from onion seed, and 12 from other hosts. All 50 *P. ananatis* strains were unable to grow on NA media amended with 200 ppm CuSO_4_^*^5H_2_O. Confluent growth was observed on all non-amended NA plates (Table 1).

### Whole genome sequencing summary

**Figure 2:**
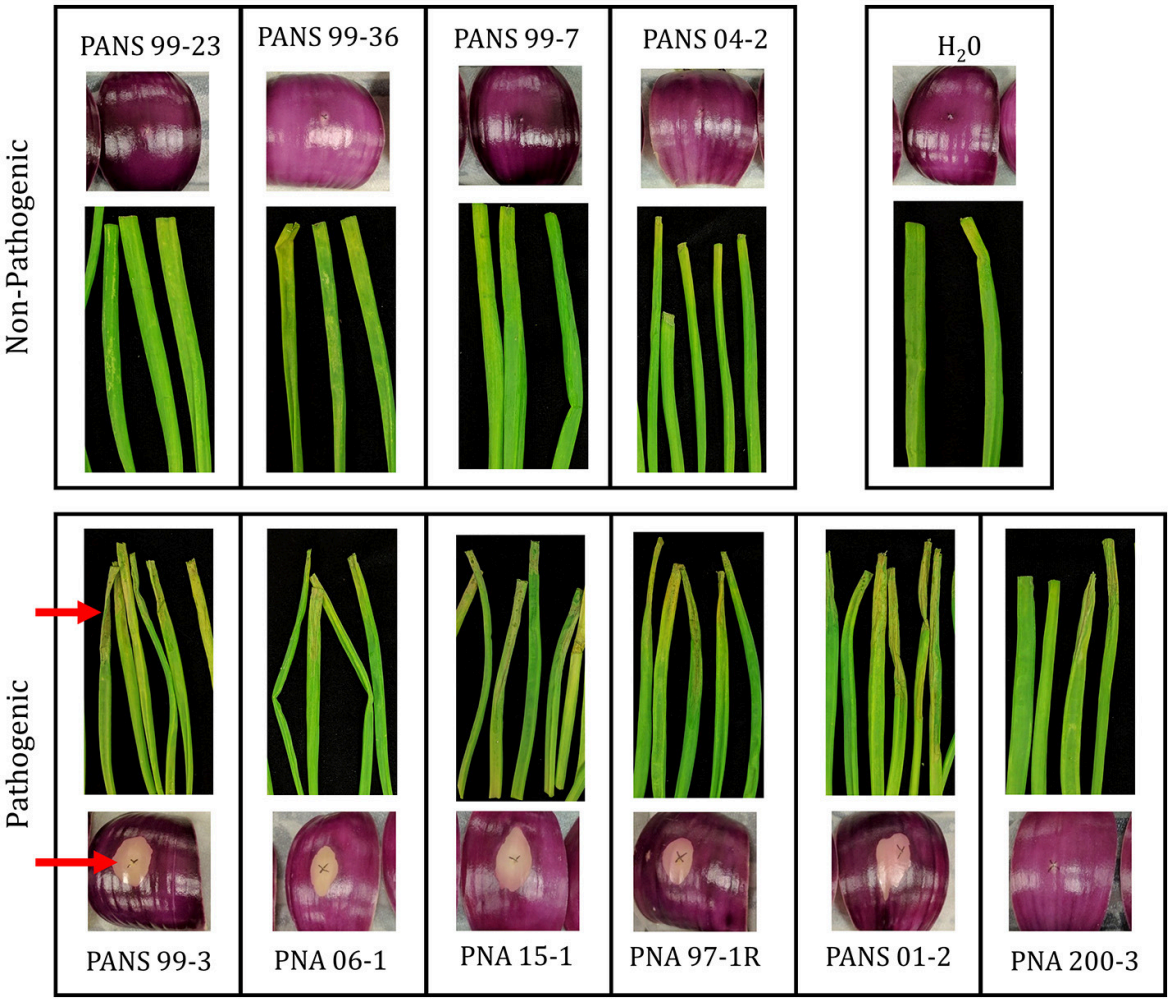
Pathogenicity of *Pantoea anantis* strains on red onion scale and onion leaves. Pathogenic strains cause clearing of pigmentation and associated weakening of onion scale. Pathogenic strains in foliar assay cause severe wilting and chlorosis along the length of the leaf blade. Blade and onion scales were inoculated with 20 uL suspension containing 1 × 10^6^ CFU/ml for each isolate. Scales were imaged 3 days post inoculation. Leaves were imaged 5 days post inoculation (Table 1). Red arrows highlight foliar wilting and scale clearing among pathogenic strains.

The prototype strain PNA 97-1R was sequenced and assembled to near completion via PacBio sequencing. In addition, whole genome Illumina shotgun sequences (WGS) of PNA 97-1R and nine additional *P. ananatis* strains from diverse primary and secondary sources associated with the center rot pathosystem were assembled and annotated to generate draft genomes for comparative pan-genomic analysis. The genomes of *P. ananatis* strain ranged from 4.8 to 5.1 Mbp size which is consistent with the reported sizes *P. ananatis* isolated from other locations (De Maayer et al., 2014). Genome features are reported in **Table 3**. The pathogenicity of each strain was assessed using both foliar and bulb scale assays (Figure 2). Annotated genomes were assessed for reported virulence elements including: Acyl homoserine lactone quorum sensing system, flagellar elements, and twitching motility type IV pilli.

**Figure 3:**
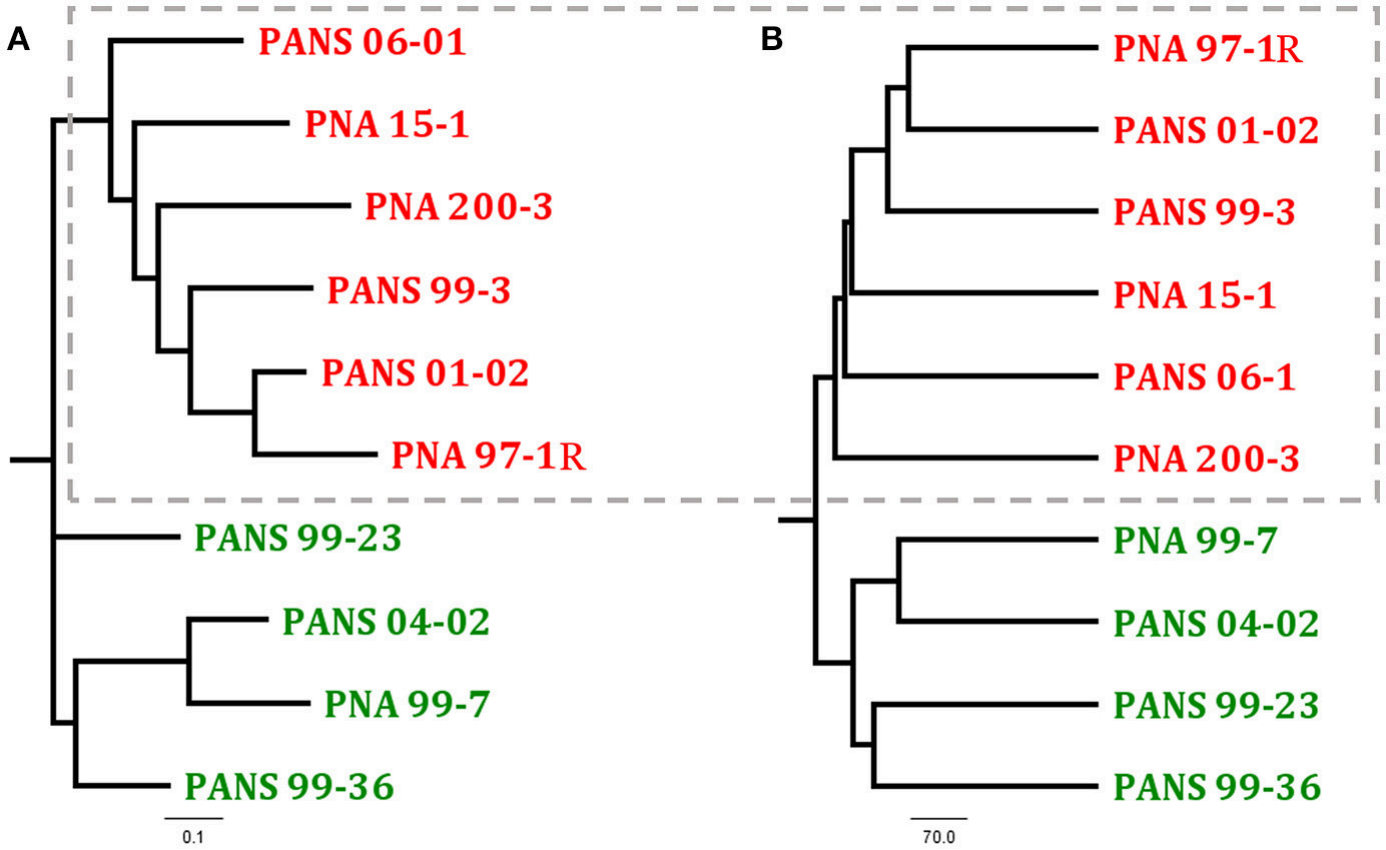
Comparison of two dendrograms of *Pantoea ananatis* strains generated through different pan-genome analysis programs **(A)** ROARY presence-absence based tree generated uses the first 4,000 genes in accessory genomes and Fast Tree to calculate distances (Page et al., 2015) **(B)** PGAP pan-genomic based UPGMA dendrogram is calculated with phylip (Zhao et al., 2012). Dotted box highlights foliar pathogen isolates. See NCBI accession numbers (Table 2).

**Figure 4:**
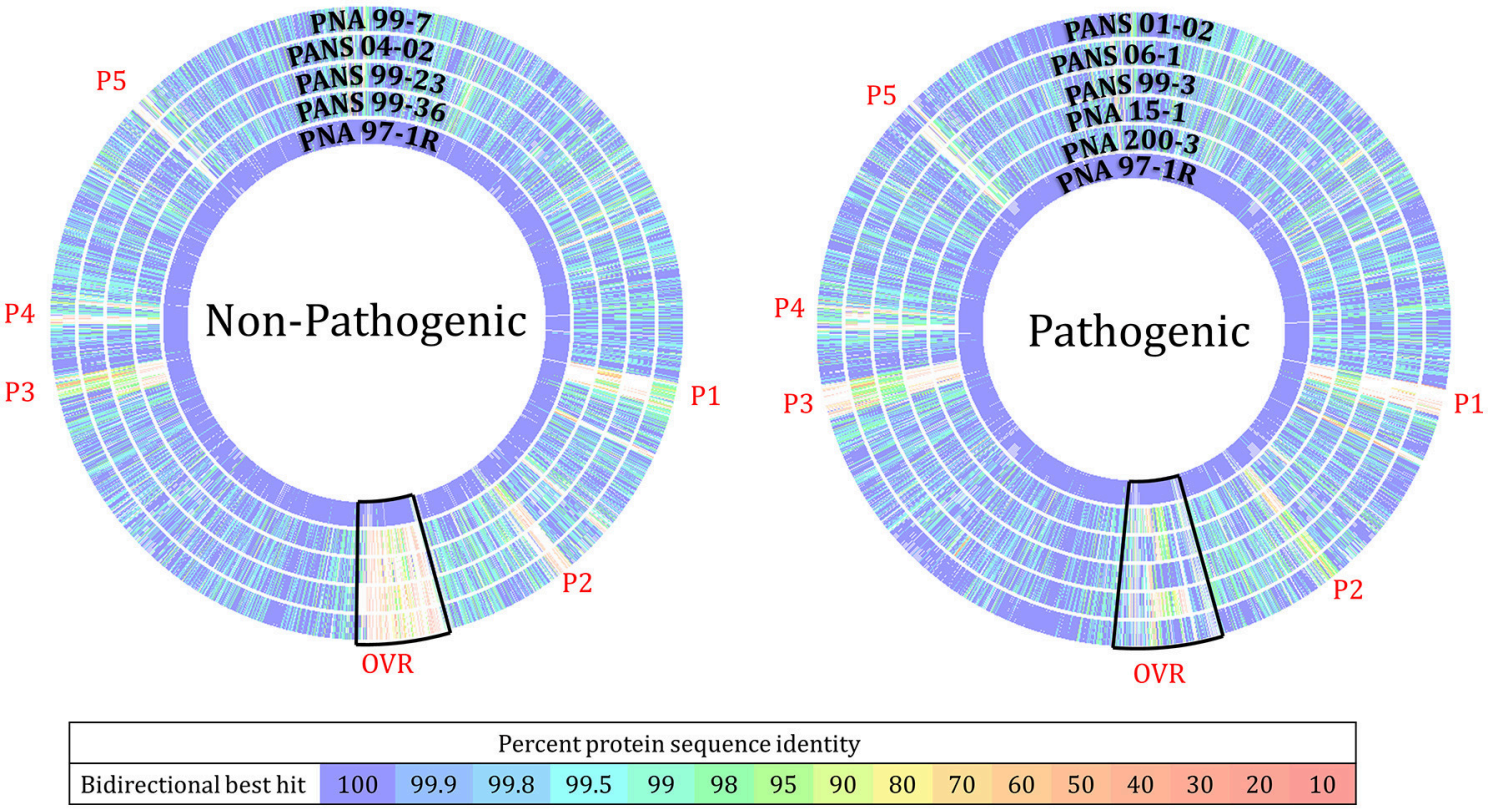
RAST Seed Viewer protein sequence-based comparison analysis of *Pantoea ananatis* strains to pathogenic proto-type strain PNA 97-1R. BLASTP analysis compares every protein in the concatenation of the reference genome (PNA 97-1R PacBio) to every protein in the comparison genome (9 other strains, Illumina HiSeq). Overlapping regions and heat map indicate level of homology between proteins. (P1) Phage related proteins including: hypothetical proteins, phage lytic proteins, phage tail proteins, phage replication proteins, phage DNA binding proteins, phage lysine and lysozymes (P2) Phage associated genes (OVR) onion virulence region loci including: cell wall degrading enzymes, metabolic related proteins, amino acid transporters, and sugar transporters. (P3) Hypothetical proteins, phage proteins, transporters, and transposable DNA elements. (P4) hypothetical proteins, and putative transcriptional regulators. This plot should not be used to determine the location of genes in the comparison genome but highlights regions that may require further analysis.

**Figure 5:**
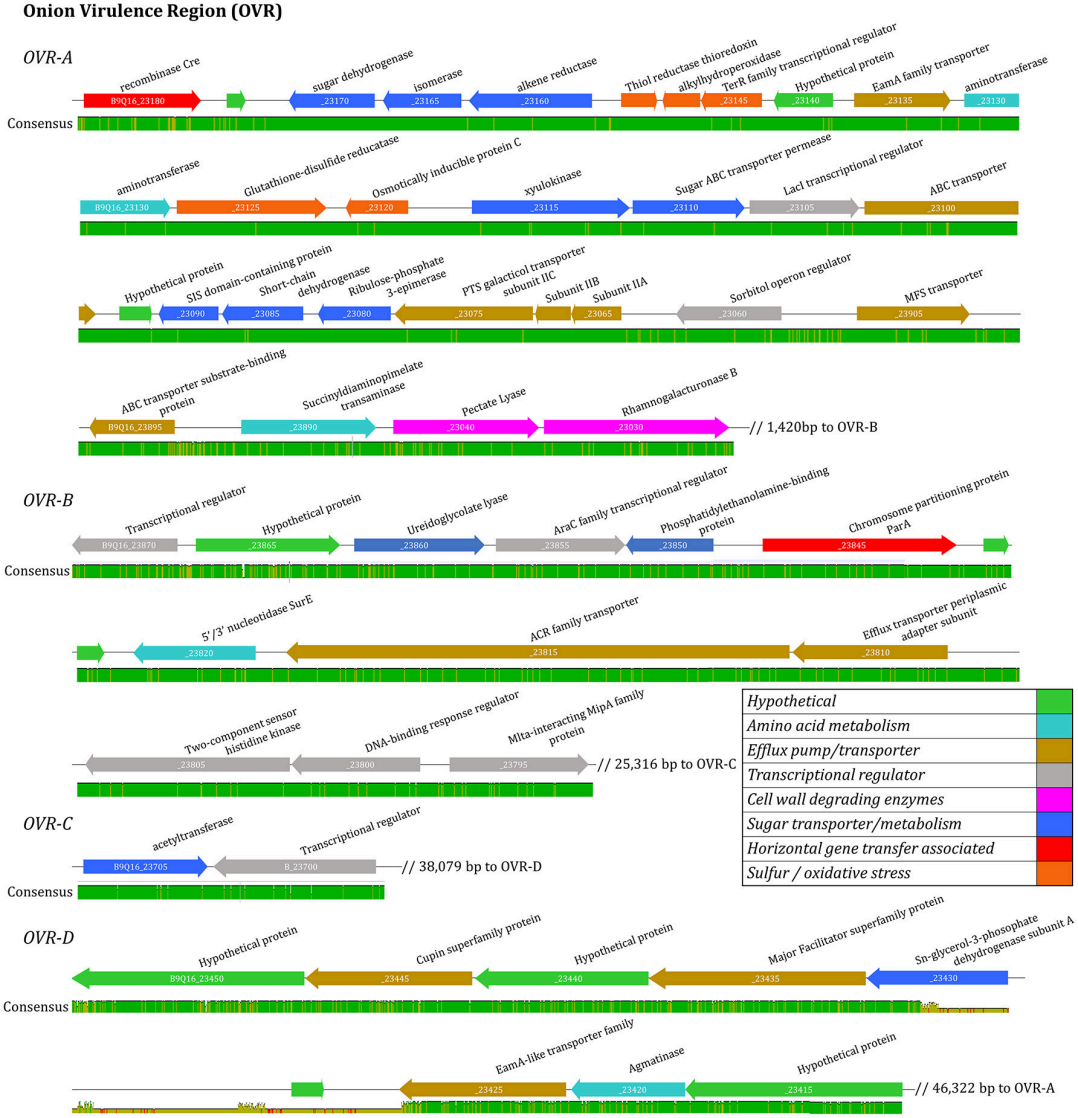
Virulence associated gene alignment for six foliar pathogenic isolates of *Pantoea ananatis*. Four clusters of virulence associated genes were identified including OVR-A, OVR-B, OVR-C, and OVR-D. The linear open-reading-frame maps of the virulence regions were identified after pan-genomic analysis and aligned using MAUVE. Genes are represented by arrows oriented in direction of transcription. Colored arrows are annotated to indicate predicated function based on manual annotation. Consensus level is illustrated with bar below ORF maps.

Universally, *P. ananatis* strains lacked complete virulence-type T3SS and T4SS secretion systems as well as the T2SS commonly used by soft rot causing Enterobacteriacea pathogens to delivery CWDEs and other virulence factors across the outer membrane. A scan of the genomes to identify NRPS/ Polyketide Synthase (PKS) clusters associated with secondary metabolites resulted in identification of clusters that encode synthetic genes for siderophores and bacteriocins. In addition, a biosynthesis cluster for the exopolysaccharide, stewartan, associated with virulence in *P. stewartii* subsp. *stewartii* was also identified (Coplin et al., 1992). Gene clusters associated with known phytotoxins were not identified. Pan-genomic analysis conducted using two pipelines with different computational strategies could differentiate onion pathogenic strains from onion non-pathogenic strains (Figure 3). Comparative analysis identified a subset of genes specific to pathogenic strains that were localized to approximately four virulence-associated regions. The largest being 31 kbp in length. We named these loci OVR-A-D- (Onion Virulence Region) due to their correlation with the onion pathogenicity (Figures 4, 5, **Table 4**).

### Red onion scale assay

The ability of *P. ananatis* strains to clear red onion scales, and cause scale weakening was observed in five of the six onion pathogenic WGS strain (Figure 2, Table 2). The weakly aggressive PNA 200-3 did not exhibit scale clearing, but did cause traditional foliar necrotic symptoms (Figure 2, Table 2). The negative control did not exhibit clearing in the onion scale assay or blighting in the foliar assay. Thus, all six strains containing the OVR-A-D region exhibited blight symptoms in foliar assay and five of six strains containing the OVR-A-D region exhibited pigment clearing in the onion scale assay (Table 2).

**Table 2:** *Pantoea ananatis* WGS strains and associated phenotypes.

**Strain Name**	**Century onion foliar assay^a^**	**Red onion scale assay^b^**	**Onion virulence region (OVR-A)^c^**	**NCBI Ascension number**	**OVR-A genomic location^d^**
PANS 99-3	++	+	+	NMZR00000000	11_7,950-38,880
PANS 99-23	−	−	−	NMZS00000000	
PANS 99-36	−	−	−	NMZT00000000	
PANS 01-2	++	+	+	NMZU00000000	9_118,393-149,370
PANS 04-2	−	−	−	NMZV00000000	
PNA 97-1R	++	+	+	CP020943-CP020945	CP020945_2,856-33,691
PNA 99-7	−	−	−	NMZW00000000	
PNA 200-3	+	−	+	NMZX00000000	11_26,412-57,631
PNA 06-1	++	+	+	NMZY00000000	11_38,312-69,184
PNA 15-1	++	+	+	NMZZ00000000	9_6,362-37,580

a *Strain ability to cause softening and pigment clearing on red onion scale 3 days post-inoculation (**Figure 2**)*.

b Strain aggressiveness on the foliage of onion. Strains lacking pathogenicity designated by “−,“ weakly aggressive “+,” highly aggressive “++.”

c *Presence or absence of Onion Virulence Region locus A based on pan-genomic analysis (ROARY & PGAP) and MAUVE alignment (Table 4, **Figure 5**)*.

d *Ordered contig with the specific location for the OVR-A region*.

### Draft genomes of 10 *P. ananatis* strains from Georgia

Quality assessment of extracted PNA 97-1R prototype strain DNA yielded fragments >50 kb. PacBio reads were assembled resulting in a high quality draft genome of prototype strain PNA 97-1R. The assembly consists of three contigs with sizes 4,571,292 bp; 295,591 bp; 180,232 bp, GC content 53.6, 52.4, and 49.6%, respectively; with an estimated 200X genome coverage (GenBank accession numbers CP020943-CP020945 respectively). The second largest contig 295,591 bp falls in the range of the large universal *Pantoea* plasmid LPP-1 280–789 kb (De Maayer et al., 2012). The presence of core factors such as thiamine biosynthesis proteins (*thiaOSF)*, and MAUVE alignment with the AJ3355 LPP-1 pEA320 (NC_017533.1) aided in characterizing this contig as homologous to the widely reported universal plasmid LPP-1 (Supplementary Figure 2; Weller-Stuart et al., 2017). MAUVE alignment yielded a single block with homology between the proposed PNA 97-1R LPP-1 plasmid (CP020944) and the AJ3355 LPP-1 plasmid (Supplementary Figure 2).

MicrobesNG Illumina draft genome sequencing yielded 354,367–2,036,787 bp range in reads for assemblies. The average insert size ranged from 344 to 649 bp (Supplementary Table 1). Average coverage for draft-genome sequences ranges from 28.9 to 167.46%. The GC content ranged from 53.29 to 53.52%. The RAST annotation resulted in prediction of 4,681–4,992 protein coding sequences (CDS) among WGS strains, roughly 1,165–1,330, were predicted as hypothetical coding sequences (Supplementary Table 2). The number of predicted phages, prophages, and transposable elements varied widely among strains from 2 to 53 CDS (Supplementary Table 2). Most proteins coding sequences are devoted to carbohydrates, amino acids and derivatives; 455–630 CDS on average. No traditional complete virulence associated plant pathogen secretion systems were identified. Two proteins associated with the type II secretion system including *gspE* (cytosolic ATPase) and *gspF* (inner membrane protein) were identified in WGS genomes of all strains.

### Known virulence factors

Virulence factors previously recorded as having importance in the pathogenicity of *P. ananatis* including: acyl-homoserine lactone mediated quorum sensing system, twitching motility, flagellar motility, two clusters of type VI secretion system (C1, C3) associated genes and effectors were present in WGS of all 10 *P. ananatis* strains. Among these, PANS 99-3 contained all three previously described type VI clusters (C1, C2, C3) (De Maayer et al., 2011).

### The ice nucleation gene (*inaZ*) is present in all strains

Although only five of the 10 WGS strains have an ice nucleation positive phenotype, the ice-nucleation protein gene *inaZ* was found to be present in all strains sequenced in this study. The *inaZ* gene was observed to vary in lengths from 3,474 bp (PANS 04-2) to 4,209 bp (PANS 06-1). PNA 200-3 has three ORFs with homology to *inaZ*s, which are severely truncated being, 750, 450, and 1,287 bp. The severely truncated nature of the *inaZ* loci in PNA 200-3 likely explains the ice nucleation minus phenotype in this strain. PANS 99-3, PNA 15-1, PNA 99-7, and PANS 04-2 encoded the *inaZ* gene but lacked an ice nucleation phenotype *in vitro*.

### Pan-genomic comparative analysis of *P. ananatis* strains from Georgia

The ROARY pan genomic analysis identified a core genome consisting of 3,750 protein coding sequences (CDS) and an accessory genome consisting of 1,395 CDS. Dendrograms generated through pan-genomic pipelines based on presence or absence of genes, differentiated onion pathogenic strains from onion non-pathogenic strains (Figure 3). ROARY BLASTP comparisons clearly identified CDS with homology to the prototype strain PNA 97-1R while also indicating phage, and virulence associated regions absent from onion non-pathogenic strains, but present in onion pathogenic strains (Figure 4, Supplementary Figure 6). Comparative analysis identified 57 CDS on four contiguous gene clusters on one contig in all pathogenic strains, but absent in onion non-pathogenic strains (Supplementary File 1). wgMLST analysis resulted in several different clades with the outgroups, *P. agglomerans* and *P. stewartii* subsp. *indologens* clustering from the *P. ananatis* strains (Supplementary Figure 4). Strains PANS 99-36 and PANS 99-23 clustered from other strains and had similar years of isolation. The pathogenic strain PANS 99-3 and non-pathogenic strain PNA 99-7 clustered together. Genomic islands were identified in all *P. ananatis* strains. Strains had between 9 and 15 predicted genomic islands. The size of the islands varied from 5,430 to 150,125 bp (Supplementary Table 4). The described OVR A-D regions were predicted as genomic islands in pathogenic strains. Islands unique to non-pathogenic strains were not identified. Partial and complete phage regions were identified in all WGS strains. The relative orientation and location of 5 phage regions can be observed in the type strain PNA 97-1R (Supplementary Figure 5).

### Identification of genomic regions associated with onion pathogenic strains

The 57 CDS shared between onion foliar pathogen strains were present on four contiguous regions, and were localized on a single contig among pathogenic strains (Table 2). The contiguous regions contained 30, 16, 2, and 9 CDS and were ordered based on orientation in the prototype strain PNA 97-1R PacBio genome (CP020945). Of the 57 CDS, 17 CDS are predicted, hypothetical proteins based on NCBI PGAP annotations (**Table 4**). The onion virulence associated regions were termed onion virulence regions A-D (OVR-A through OVR-D) (**Table 4**, Figure 5). All four regions are located on the smallest 180,232 bp contig from PNA 97-1R PacBio assembly (CP020945). PGAP pan-genomic analysis, which utilizes computationally intensive strategy to BLAST every gene to each genome confirmed, 20 of the pathogen associated genes were present only once in the onion pathogenic strains and entirely absent in the non-pathogenic strains (**Table 4**, Figure 5). MAUVE alignment of pathogenic strains along these regions indicated high levels of homology (Figure 5).

The OVR-A locus begins with a gene for a *Cre-*like phage recombinase, which may indicate integration of the 31-kb OVR-A locus as a mobilized genomic island. A gene closely following *Cre* is predicted to be a TetR family transcriptional regulator. Closely following this regulator are sugar transport proteins including a sugar ABC transporter permease and ribulose-phosphate-3-epimerase. The OVR-A contains three genes associated with sulfur, redox and oxidative stress, such as a thiol reductase and alkylhydroperoxidase. Several efflux pumps and transporters are present including an ABC transporter and PTS galacticol transporter subunit IIA-C. Amino acid metabolism-transport and plant cell wall degrading enzymes (CWDE) including a pectate lyase, and rhamnogalacturoase B are also present in the OVR-A region (**Table 4**, Figure 5).

OVR-B consists of 16 CDS. Three proteins are predicted to have DNA-binding regulatory functions such as the AraC family transcriptional regulator. A ureidoglycolate lyase is associated with amino acid degradation. Two outer membrane transport proteins are present including an ACR family transporter. Two protein kinases are present including a predicted two-component sensor histidine kinase and a phosphatidylethanolamine-binding protein with a predicted secretion target signal. In addition, three proteins are present related to transposable DNA elements including an IS*110* family transposase. An Mlta-interacting family protein lytic transglycosylases, which is related to protein binding membrane, is also present (**Table 4**).

OVR-C contains a transcriptional repressor with homology to *PuuR*, and an acetyl-transferase *YjaB* (**Table 4**). OVR-D contains nine predicted CDS. The PGAP annotation pipeline characterized all as hypothetical proteins. Porkka annotation predicted two transport associated proteins including a Major Facilitator Superfamily CDS (**Table 4**). In addition, two proteins involved in carbon and amino acid metabolism, including agmatinase are also present.

Several of the CDS in the regions specific to onion pathogenic strains have predicted transmembrane or Tat-secreted target signal. CDS of interest with Tat-secreted target signals include: CWDE pectate lyase, and rhamnogalacuroase in the OVR-A and two protein signal regulators in OVR-B. Most proteins predicted to be transport-related contained transmembrane domains (**Table 4**).

## Discussion

### Phenotypic variation amongst *P. ananatis* strains

A set of fifty *P. ananatis* strains from Georgia was assembled to maximize diversity of host and geographic sources of isolation as well as diversity of previously determined onion pathogenicity and ice nucleation phenotypes. We observed extensive phenotypic variation among a sub-set of 33 strains tested for pathogenicity and aggressiveness on multiple *Allium* hosts. In addition to the 15 primary onion and onion seed strains, 10 of 25 PANS strains collected from weed and thrips species were re-confirmed to be pathogenic on onion. However, aggressiveness of strains on *Allium* sp. varied considerably. Among the 33 strains, we observed similar levels of variation in pathogenic potential and aggressiveness on shallot and leek although the strains were generally less aggressive on these hosts than on onion. Pathogenic potential and aggressiveness of a strain on onion or leek or shallot did not generally correspond with its potential to be pathogenic on the other *Allium* hosts. In addition, there were multiple examples of onion non-pathogenic strains that were pathogenic on leek or shallot and vice versa such as PANS 99-23. This could indicate some degree of host specialization among *P. ananatis* strains tested. Unlike other *Allium* hosts, the chive cv. Dolores was infected by only a single *P. ananatis* strain with mild symptoms. This may highlight the potential *of Allium schoenoprasum* to serve as a source of genetic resistance against *P. ananatis*.

We used MLSA and rep-PCR based genetic diversity analysis to determine whether phenotypic diversity correlated with underlying phylogeny. Contrary to the high phenotypic variation, both analyses indicated little genetic diversity amongst the fifty *P. ananatis* strains. By MLSA all strains strain were found to be >99% identical. The majority of branches in the phylogeny had poor bootstrap value support and were composed of a mixture of PNA and PANS strains. We observed no underlying correlation between the isolate phylogeny and pathogenic phenotypes or isolation sources using either method of diversity analysis.

Motivated by the high degree of *P. ananatis* phenotypic variation that is unexplained by the low levels of core genome diversity, we sought to conduct a pan-genome analysis to determine whether fine scale variations in gene presence-absence might correlate with phenotypic variation. A sub-set of 10 strains from the original fifty strains were selected for whole genome Illumina shotgun sequencing with the goal of maximizing diversity of phenotypes and sources of isolation. Additionally, the Georgia prototype onion pathogenic strain PNA 97-1R was sequenced using PacBio to create a high quality near-closed genome assembly. Among the 10 strains selected for WGS, six were able to cause foliar necrosis in onion blades and five of those also were also able to clear red onion scales (Figure 2). The phenotypic variation seen in red-onion scale assays may potentially be a useful tool for identifying isolates with onion pathogenic potential as it is easy to replicate and rapid with minimal space requirements. It would be beneficial to utilize more strains to determine if correlations between red onion scale clearing and onion foliar blight potential occur.

### Pan-genome analysis of sequenced isolates

Dendrograms generated from pan-genomic gene presence-absence analysis clearly indicate a clustering of onion pathogenic strains from non-pathogenic strains. This suggests an underlying genetic difference between the two groups that was not detected using MLSA or rep-PCR approaches. The majority of the gene differences between onion pathogenic and non-pathogenic strains resides in four contiguous gene clusters the largest being 31 kb that we termed as the Onion Virulence Region (OVR-A). RAST BlastP further illustrated this point by identifying genes present in onion pathogenic strains, but absent in non-pathogenic strains when compared with the pathogenic prototype strain PNA 97-1R. The level of core genome homology amongst all the strains is notable. There is a high degree of CDS similarity. Most gene variation is the result of phage, transposase, and integrase proteins. In addition, homologs to the Lux1, type VI, V, I s, and Tat secretion systems previously noted as candidate virulence factors were identified in both onion pathogenic and non-pathogenic strains. Phytotoxin synthetic clusters were not identified (Weller-Stuart et al., 2017).

The *de-novo* genome assembly of the Georgia prototype strain PNA 97-1R resulted in three contigs, of 4.9 Mbp, 295 kbp, and 180 kbp (CP020943-CP020945). The 4.9 Mbp contig corresponds to the *Pantoea* circular chromosome. The 295 kbp contig was identified as the Large Pantoea Plasmid LPP-1 based on previously described genes and MAUVE alignment. The genomes of the sequenced strains contain LPP-1 with a high level of conservation (Supplementary Figure 3). The third 180 kbp PNA 97-1R contig encompasses the four OVR loci which correlate with onion pathogenicity based on pangenomic analysis. This contig aligned with single contigs of varying sizes (74–167 kbp) from onion pathogenic WGS strains. The 180 kbp contig shares complete homology in 6 MAUVE alignment blocks with all genes in a 60 kbp contig (CAEI01000108.1) of a single sequenced strain B1-9 of *P. ananatis* in the NCBI database (NZ_CAEI00000000.1). Strain B1-9 is an isolate from green onion described by researchers in South Korea associated with growth promotion of pepper (Kim et al., 2012). It is intriguing that a growth promoting strain from South Korea share all genes associated with onion virulence among our Georgia strains. We know that the pathogenicity of *P. ananatis* strains can vary widely between different species of *Allium* (Table 1). It would be interesting to determine if B1-9 is pathogenic on onion and on other *Allium* sp. In addition to sharing homology with this contig from B1-9, the 180 kbp contig shares some genes with those found on the pEcWSU1 plasmid of the onion pathogen, *Enterobacter cloacae* associated with onion bulb-rot (Schroeder et al., 2009). Highest homology is shared between a histidine kinase protein in OVR-B, and the PTS galacticol transporter system A-C in the OVR-A loci. Interestingly, plasmid partitioning genes and repA plasmid replication genes were present on the WGS contigs that contained the OVR-A loci in each of the onion pathogenic strains indicating that these contigs may, in fact, represent an accessory plasmid.

*Pantoea ananatis* is a species that is frequently associated with diverse eukaryotic hosts. In the onion-center rot system, it is associated epiphytically with weed species, and pathogenically and endophytically with onions, and persists temporarily in the thrips vector commonly associated with center-rot disease outbreaks (Gitaitis et al., 2002; Walcott et al., 2002; Dutta et al., 2014). Our strains were isolated from various sources such as thrips, weeds, seeds, and symptomatic onions. Despite these strains inhabiting different niches, there existed a high degree of genetic similarity among them. Furthermore, a high average nucleotide identity was observed among Georgia isolates and other strains isolated in different regions around the world (Table 3). This suggests that the core genome is highly conserved among *P. ananatis* strains (Supplementary Figure 3). The core genome of 3,750 CDS identified through ROARY pan-genomic analysis is consistent with the core genome identified through previous pan-genomic analysis methods (De Maayer et al., 2014; Sheibani-Tezerji et al., 2015). The accessory genome, where most differences were identified, revealed the Onion Virulence regions (OVR-AD), as well as differences among poorly characterized hypothetical coding sequences, phages, transposases, integrases, and mobile genetic elements. The presence of the *Cre* recombinase in the OVR-A loci could be indicative of a possible horizontal gene transfer mechanism.

**Table 3:** Primers and conditions used for amplification and sequencing.

**Gene**	**Primer name**	**Primer sequence**	**Primer positions on coding sequences**	**PCR cycles**	**Template size (bp)**	**Template position on *P. ananatis* PA13 genome**
fusA	fusA3	5′-CAT CGG TAT CAG TGC KCA CAT CGA-3′	36–59	2 min 94°C; 1 min 94°C 1 min 58°C 1 min 72°C (31 cycles); 5 min 72°C	639	4449773–450416
	fusA4	5′-CAG CAT CGC CTG AAC RCC TTT GTT-3′				
gyrB	gyrB3	5′-GCG TAA GCG CCC GGG TAT GTA-3′	57–77	2 min 94°C; 1 min 94°C 1 min 58°C 1 min 72°C (31 cycles); 5 min 72°C	427	4108–4543
	gyrB4	5′CCG TCG ACG TCC GCA TCG GTC AT-3′	1488–1508			
	gyrB3i	5′-AAC GCW ATC GAC GAA GC-3′	136–152	Primers used only for sequencing		
	gyrB4i	5′-TGG AAC CCR TCR TTC CAC-3′	771–788			
leuS	leuS3	5′-CAG ACC GTG CTG GCC AAC GAR CAR GT-3′	487–512	2 min 94°C; 1 min 94°C 1 min 58°C 1 min 72°C (31 cycles); 5 min 72°C	640	3282463–3283102
	leuS4	5′-CGG CGC GCC CCA RTA RCG CT-3′	1274–1293			
pyrG	pyrG3	5′-GGG GTC GTA TCC TCT CTG GGT AAA GG-3′	31–56	2 min 94°C; 1 min 94°C 1 min 58°C 1 min 72°C (31 cycles); 5 min 72°C	316	1019655–1019970
	pyrG4	5′-GGA ACG GCA GGG ATT CGA TAT CNC CKA-3′	434–460			
rplB	rplB3	5′-CAG TTG TTG AAC GTC TTG AGT ACG ATC C-3′	227–254	2 min 94°C; 1 min 94°C 1 min 58°C 1 min 72°C (31 cycles); 5 min 72°C	343	457307–457649
	rplB4	5′-CAC CAC CAC CAT GYG GGT GRT C-3′	685–706			
rpoB	Vic3	5′-GGC GAA ATG GCW GAG AAC CA-3′	1422–1442	4 min 94°C; 30 s 94°C 30 s 50°C 30 s 72°C (31 cycles); 5 min 72°C	596	4432827–4433422
	Vic2	5′-GAG TCT TCG AAG TTG TAA CC-3′	2469–2489			

*^a^Size of the sequence template of the internal portion of PCR product used for sequence comparison*.

*^b^Location of each gene sequence analysis on P. ananatis PA 13 reference genome (NCBI genome accession no. CP003085)*.

### Genetic content of the OVR-A loci

The OVR-A loci has not been previously described and may offer insights into the ability of *P. ananatis* to cause bacterial disease in onions. This region was present in all Georgia strains that caused the foliar symptoms on onion (Figure 2). Except for strain PNA 200-3, this region was present in all *P. ananatis* strains that caused scale clearing in the red onion scale assay. The region begins with a *Cre* recombinase followed by amino acid transporters, metabolic enzymes, transcriptional regulators and CWDEs (Table 4).

**Table 4:** ROARY presence-absence based comparison.

**Gene**	**Prokka annotation**	**NCBI locus**	**NCBI PGAP annotation**	**Length (bp)**
		**PNA 97-1R**		
**ONION VIRULENCE REGION A (OVR-A)**				
*xerC*	Tyrosine recombinase XerC	B9Q16_23180	Recombinase Cre	1,038
	Helix-turn-helix	B9Q16_23175	Hypothetical protein CDS	213
fabG^*^	3-oxoacyl-[acyl-carrier-protein] reductase FabG	B9Q16_23170	Sugar dehydrogenase CDS	774
	DSBA-like thioredoxin domain	B9Q16_23165	Isomerase CDS	702
*nemA*	N-ethylmaleimide reductase	B9Q16_23160	Alkene reductase CDS	1,116
	Thioredoxin-like protein slr0233	B9Q16_23155	Thiol reductase thioredoxin CDS	354
	Carboxymuconolactone decarboxylase family	B9Q16_23150	Alkylhydroperoxidase CDS	339
*nemR*	HTH-type transcriptional repressor NemR	B9Q16_23145	TetR family transcriptional regulator	579
	hypothetical protein	B9Q16_23140	Hypothetical protein CDS	528
*yijE^Tr^*	Uncharacterized inner membrane transporter yiJE	B9Q16_23135	EamA family transporter CDS	864
*patB*	Cystathionine beta-lyase PatB	B9Q16_23130	Aminotransferase CDS	1,212
*gor*	Glutathione reductase	B9Q16_23125	Glutathione-disulfide reductase CDS	1,356
*osmC*	OsmC-like protein	B9Q16_23120	Osmotically inducible protein C CDS	558
*xylB*	Xylulose kinase	B9Q16_23115	Xylulokinase CDS	1,449
*rbsC^*Tr*^*	Ribose transport system permease protein RbsC	B9Q16_23110	Sugar ABC transporter permease CDS	1,026
*rbsB*	D-ribose-binding periplasmic protein precursor	B9Q16_23105	LacI family transcriptional regulator CDS	981
*rbsA*	Ribose import ATP-binding protein RbsA	B9Q16_23100	ABS transporter CDS	1,503
	Hypothetical protein	B9Q16_23095	Hypothetical protein CDS	285
*hxlB*	3-hexulose-6-phosphate isomerase	B9Q16_23090	SIS domain-containing protein	597
*fabG*	3-oxoacyl-[acyl-carrier-protein] reductase FabG	B9Q16_23085	Short-chain dehydrogenase CDS	753
*Rpe*	Ribulose-phosphate 3-epimerase	B9Q16_23080	Ribulose-phosphate 3-epimerase CDS	645
*gatC^*Tr*^*	Galactitol permease IIC component	B9Q16_23075	PTS galacticol transporter subunit IIC CDS	1,254
*gatB^*Tr*^*	Galactitol-specific phosphotransferase enzyme IIB component	B9Q16_23070	PTS galacticol transporter subunit IIB CDS	294
*fruA*	PTS system fructose-specific EIIABC component	B9Q16_23065	PTS galacticol transporter subunit IIA CDS	450
*sorC*	Sorbitol operon regulator	B9Q16_23060	Hypothetical protein CDS	969
	Major Facilitator Superfamily	B9Q16_23905	MFS transporter CDS	1,143
*argT*	Lysine-arginine-ornithine-binding periplasmic protein precursor	B9Q16_23895	ABC transporter substrate-binding protein CDS	783
*dapL*	LL-diaminopimelate aminotransferase	B9Q16_23890	Succinyldiaminopimelate transaminase CDS	1,203
pelE^*^	Pectate lyase E precursor	B9Q16_23040	Pectate lyase CDS	1,194
^*^	Rhamnogalacturonase B, N-terminal	B9Q16_23030	Rhamnogalacturonase B CDS	1,610
**ONION VIRULENCE REGION B (OVR-B)**				
csiR^*^	HTH-type transcriptional repressor CsiR	B9Q16_23870	Transcriptional regulator CDS	693
	Major royal jelly protein	B9Q16_23865	Hypothetical protein CDS	930
	Ureidoglycolate lyase	B9Q16_23860	2-hydroxyhepta-2,4-diene-1,7-dioate isomerase CDS	840
*ydeO*	HTH-type transcriptional regulator YdeO	B9Q16_23855	AraC family transcriptional regulator CDS	828
^*^	putative kinase inhibitor	B9Q16_23850	Phosphatidylethanolamine-binding protein CDS	564
	T5orf172 domain	B9Q16_23845	Chromosome partitioning protein ParA CDS	1,564
	hypothetical protein	B9Q16_23840	IS*110* family transposase	207
	hypothetical protein	B9Q16_23835	Hypothetical protein CDS	192
	hypothetical protein	B9Q16_23830	Hypothetical protein CDS	978
*surE*	5′-nucleotidase SurE	B9Q16_23825	5′/3′ nucleotidase SurE	777
*slyA*	transcriptional regulator SlyA	B9Q16_23820	Hypothetical protein CDS	426
*acrF*	Multidrug export protein AcrF	B9Q16_23815	ACR family transporter CDS	3,084
bepF^*^	Efflux pump periplasmic linker BepF	B9Q16_23810	Efflux transporter periplamsic adapter subunit CDS	1,146
baeS^*^	Signal transduction histidine-protein kinase BaeS	B9Q16_23805	Two-component sensor histidine kinase CDS	1,170
*phoP*	Alkaline phosphatase synthesis transcriptional regulatory protein PhoP	B9Q16_23800	DNA-binding response regulator CDS	714
*mipA*	MltA-interacting protein MipA	B9Q16_23795	Mlta-interacting MipA family protein CDS	783
**ONION VIRULENCE REGION C (OVR-C)**				
*yjaB*	Uncharacterized N-acetyltransferase YjaB	B9Q16_23705	Acetyltransferase	444
*puuR*	DNA-binding transcriptional repressor PuuR	B9Q16_23700	Transcriptional regulator CDS	585
**ONION VIRULENCE REGION D (OVR-D)**				
	Major Facilitator Superfamily	B9Q16_23450	Hypothetical protein CDS	1,251
	Hypothetical protein	B9Q16_23445	Hypothetical protein CDS	903
	Cupin superfamily protein	B9Q16_23440	Hypothetical protein CDS	930
	Hypothetical protein	B9Q16_23435	Hypothetical protein CDS	1,170
	Hypothetical protein	B9Q16_23435	Hypothetical protein CDS	1,170
*speB*	Agmatinase	B9Q16_23430	Hypothetical protein CDS	1110
*eamA*	EamA-like transporter family	B9Q16_23425	Hypothetical protein CDS	880
	Hypothetical protein	B9Q16_23420	Hypothetical protein CDS	618
	sn-glycerol-3-phosphate dehydrogenase subunit A	B9Q16_23415	Hypothetical protein CDS	1,134

*Genes identified in Georgia pathogenic strains, and absent in non-pathogenic strains of Pantoea ananatis*.

Tr *Predicted Transmembrane protein (TMHMM v 2.0)*.

* *Tat/ Sec Secreted target signal present (Signal P 4.1)*.

The presence of redox-related/sulfur reducing enzymes could play a role in withstanding host sulfur containing antimicrobials, or interrupting ROS signaling that is important during plant immunity (Torres et al., 2006). In addition, amino acid transporters may play a role in the absorption of host nutrients. The specific localization of *P. ananatis* in host tissue has not been well characterized and may offer insights into nutrient acquisition for this bacterium (Fatima and Senthil-Kumar, 2015). Sugar transporters and major super facilitator proteins have been shown to play an important role in the pathogenicity of bacterial pathogens such as *Dickeya dadantii* where they can play a role in the anti-microbial resistance, and chemotaxis (De Maayer et al., 2011).

Plant cell wall degrading enzymes such as *pelE* and rhamnogalacturonase, have been found to contribute to pathogenicity in a number of other bacterial pathogens, notably *Dickeya dadantii* (Hassan et al., 2013). A protein in OVR-D was computationally annotated as a member of the *EamA* transport family shares homology with the *PecM*, a transporter in *D. dadantii*, which plays a major role in regulating virulence factor synthesis such as, *PelE* and other CWDEs (Praillet et al., 1996). However, as *P. ananatis* lacks a T2SS it is unclear by what mechanism these proteins would be secreted out of the cell. The protein kinase and kinase inhibitor found in OVR-B are intriguing as they both have predicted secretion signals (putative kinase inhibitor, two-component sensor histidine kinase) (Table 4). They could play a role in signal transduction in the bacteria or perhaps could be involved in interrupting host immunity by some as-of-yet uncharacterized host cell delivery mechanism. Transporter and transmembrane proteins located in onion virulence correlated regions could be involved in secretion of proteins across the outer membrane, or surface interactions between the bacteria and host cells.

The localization of OVR A-D to single contigs from the genome constructs offers some supportive evidence that the region is plasmid localized. Symbiotic and pathogenic plasmids are important for pathogenicity in a number of phytopathogenic bacterial species. In plant pathogens mega plasmids have been shown to contribute to virulence in bacteria such as *Pseudomonas syringae* pv. *phaseolicola* (Jackson et al., 1999). The contigs constructs containing OVR locci contain parA plasmid partitioning proteins. In our future work work we will seek to confirm whether the OVR loci are, in fact, plasmid-borne.

Few pan-genomic analyses of *P. ananatis* strains have been conducted. The first pan-genomic study of *P. ananatis* utilized NCBI draft and complete genomes of strains isolated from various hosts. It is difficult to infer the genomic diversity of strains isolated from a single geographic location or host-pathosysem from such, studies, as hosts, isolation sources, and locations varied dramatically (De Maayer et al., 2014). Currently, only one study elucidated the genomic variation among *P. ananatis* strains, isolated from the same host, maize seed. The researchers observed a high degree of genetic similarity among *P. ananatis* strains with diverse phenotypes; epiphytic, plant growth-promoting, and pathogenic (Sheibani-Tezerji et al., 2015). Despite high levels of genetic similarity, minute differences in genes encoding protein secretion systems, putative effectors, and transpose/integrases/phage related genes were noted. For example, the pathogenic *P*. *ananatis* genome contained genes relating to cell cycle control, cell division, chromosome partitioning, and amino acid transport that differentiated it from the growth promoting, and endophytic strain. This study found subtle variations between the pathogenic and non-pathogenic strains. Other protein coding sequences that differed between the strains included transposase related proteins, chemotaxis proteins, and T6SS loci (Sheibani-Tezerji et al., 2015). Conversely, our results identified a distinct subset of genes in contiguous regions that correlated with onion pathogenicity.

The presence of genes within the OVR-A loci associated with virulence and pathogenicity in other onion pathogens such as *Enterobacter cloacae* illustrates a need to further characterize genes in this region and other onion virulence correlated regions. Future work will include the deletion of the OVR-A loci from PNA 97-1R to determine what role this region plays in onion pathogenicity.

Strain PNA 200-3 has the OVR-A region but lacks the scale clearing phenotype. The negative-scale clearing phenotype may be explained by SNP missense mutations in genes encoding CWDEs of PNA 200-3's OVR-A or could indicate variation in expression of genes associated with this loci. The OVR-A loci may simply represent a vertically inherited locus or plasmid common to Georgia onion pathogenic strains through a common clonal origin of onion pathogenic strains in Georgia, and possible relation to strains in South Korea. Thus, while the OVR-A loci is common among Georgia onion pathogenic strains, it may not in itself, carry pathogenicity determinants that are critical to infection. Many other currently sequenced strains have been reported as pathogenic on onion, while apparently lacking the OVR loci (Morohoshi et al., 2007; Shyntum et al., 2015).

## Conclusions

Overall, our study illustrates the importance of WGS in differentiating closely related strains. MLSA and rep-PCR analysis indicated little diversity among the *P. ananatis* strains in Georgia, although the strains were phenotypically diverse for *Allium* infection phenotypes. The WGS revealed that although these strains share similar core genomes, the universal plasmid LPP-1, and previously identified candidate virulence factors, differences in the mobile genetic elements, as well as the OVR-A loci differentiated the sequenced strains into two groups that shared common scale-clearing and foliar pathogenicity phenotypes. Thus, the differences between pathogenic and non-pathogenic strains in *P. ananatis* may not be as subtle as previously suggested (Sheibani-Tezerji et al., 2015). In any case, the contribution of the OVR loci to *P. ananatis* onion virulence remains to be determined. Subtle differences in loci gene expression and host defense reactions may further differentiate host range and aggressiveness of *P. ananatis* strains. The transcriptional analysis of mutants with and without these loci may provide insights into the nature of this regions contributions to virulence. Future studies will involve the use of mutational analysis to assess the role of the OVR-A loci in onion pathogenicity and determine if this genetic locus is chromosomal or plasmid-borne.

## Author contributions

SPS and SDS contributed equally to this work. BK and BD are co-senior on this work. SPS, SDS, RG, BK, and BD conceived and designed the experiments. SDS performed MLSA, rep-PCR and phenotypic testing. SPS conducted experiments and analysis related to whole genome sequencing and Pan-genome analysis. SPS, SDS, BK, and BD wrote and edited the manuscript.

### Conflict of interest statement

The authors declare that the research was conducted in the absence of any commercial or financial relationships that could be construed as a potential conflict of interest.

## Supplementary material

Supplementary Figure 1 Dendrogram derived by cluster analysis of similarities between *Pantoea ananatis* strains based on repetitive extragenic palindromic polymerase chain reaction (REP-PCR) DNA genomic profiles. Analysis was performed using Dice's (1945) coefficient in BioNumerics software package (Applied Math, Kortrijk, Belgium).

Supplementary Figure 2 Large Pantoea Plasmid LPP-1 alignment of proto-type strain PNA 97-1 with AJ13355 pEA320 (Accession CP020943; NC_017533.1) Alignment generated with Geneious MAUVE plugin.

Supplementary Figure 3 Venn diagram of genes shared among the pathogenic isolate PNA 97-1R; nonpathogenic isolates PANS 99-36, and genomic type strain LMG 20103 (Accession CP001875.2). The number of coding sequences are derived from the gene_presence_absense.csv output of the ROARY pan-genome analysis using default parameters (Supplementary File 2).

Supplementary Figure 4 wgMLST cladogram of *Pantoea* strains generated through PGAdb-builder platform. Outgroups *P. agglomerans P10C and P. stewartii* subsp. *indologens* LMG 2632 (Accession NZ_LIME00000000.1 and JPKO00000000.1).

Supplementary Figure 5 Phaster predicted phage regions in PNA 97-1R. Green indicated intact phage regions whereas red indicates incomplete phage regions.

Supplementary Figure 6 BRIG analysis of *P. ananatis* strains and the OVR A-D loci to the reference strain PNA 97-1R. BRIG is a locally run analysis that generates figures based on blastn comparisons of query genomes to a reference strain.

Supplementary Table 1 Sequencing and assembly statistics for Illumina draft-genomes of *Pantoea ananatis* strains.

Supplementary Table 2 RAST functional categories for predicted protein coding sequences of *Pantoea ananatis* strains.

Supplementary Table 3 Primers and conditions used for amplification and sequencing.

Supplementary Table 4 IslandPath DIMOB genomic islands identified in WGS *P. ananatis* strains.

Supplementary File 1 ROARY 10 strain WGS Analysis. The presence of each CDS in the respective genome is listed below each labeled strain column. If a cell is blank the respective strain does not have the gene. Logic equations were used to highlight cells, resulting in genes present in pathogenic strains and absent in non-pathogenic strains.

Supplementary File 2 ROARY 3 strain WGS Analysis. The presence of each CDS in the respective genome is listed below each labeled strain column. If a cell is blank the respective strain does not have the gene. Summation equations were used to calculate the numeric values for the Venn Diagram in Supplementary Figure 3.
