# Supplementary figures and images for: Pantoea ananatis Genetic Diversity Analysis Reveals Limited Genomic Diversity as Well as Accessory Genes Correlated with Onion Pathogenicity
Source: Front Microbiol. 2018 Feb 13;9:184. doi: 10.3389/fmicb.2018.00184 (PMC5817063; doi:10.3389/fmicb.2018.00184)

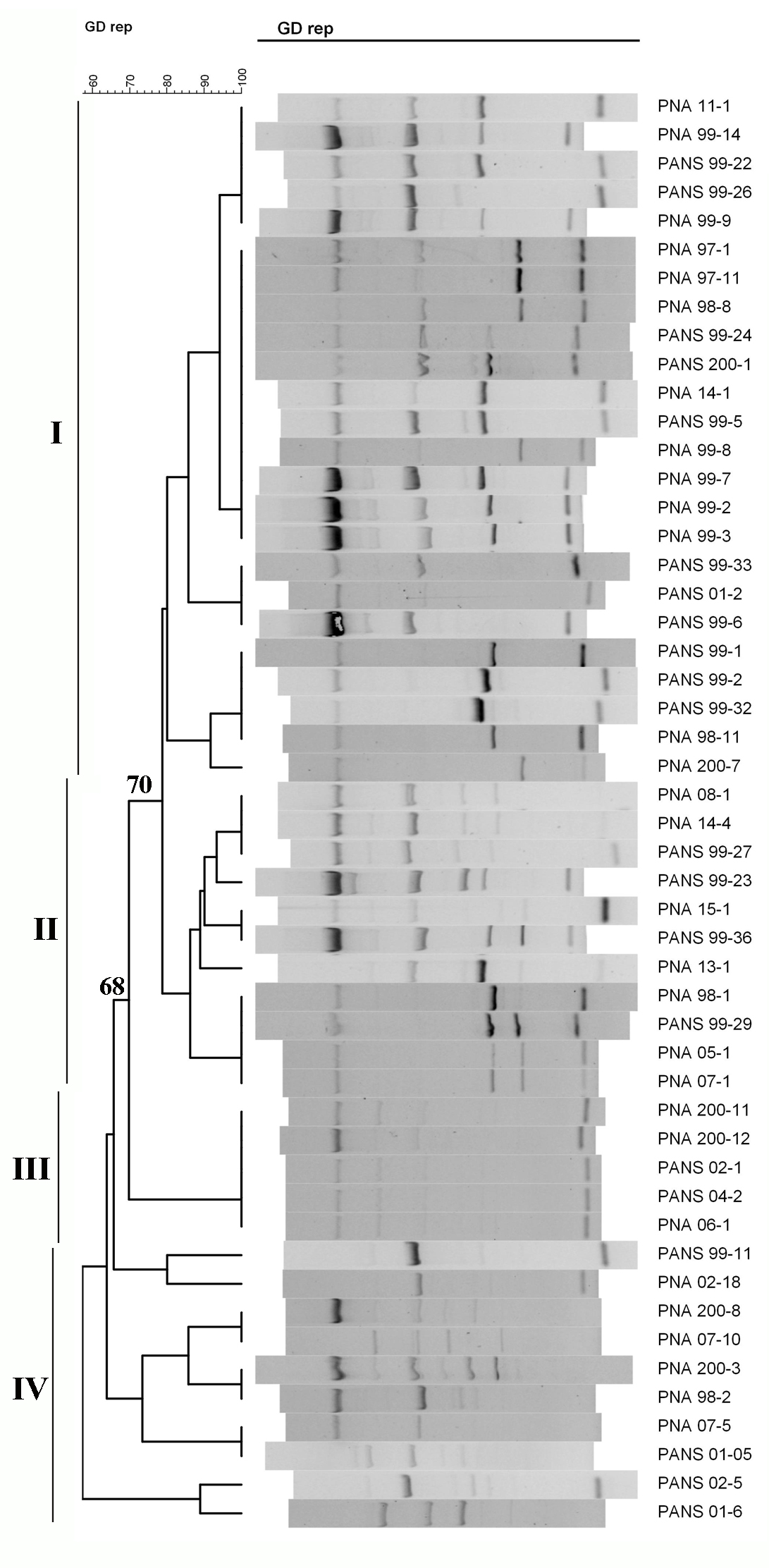

Supplement: Supplementary Figure 1 — Dendrogram derived by cluster analysis of similarities between Pantoea ananatis strains based on repetitive extragenic palindromic polymerase chain reaction (REP-PCR) DNA genomic profiles. Analysis was performed using Dice's (1945) coefficient in BioNumerics software package (Applied Math, Kortrijk, Belgium). [file Image1.JPEG]

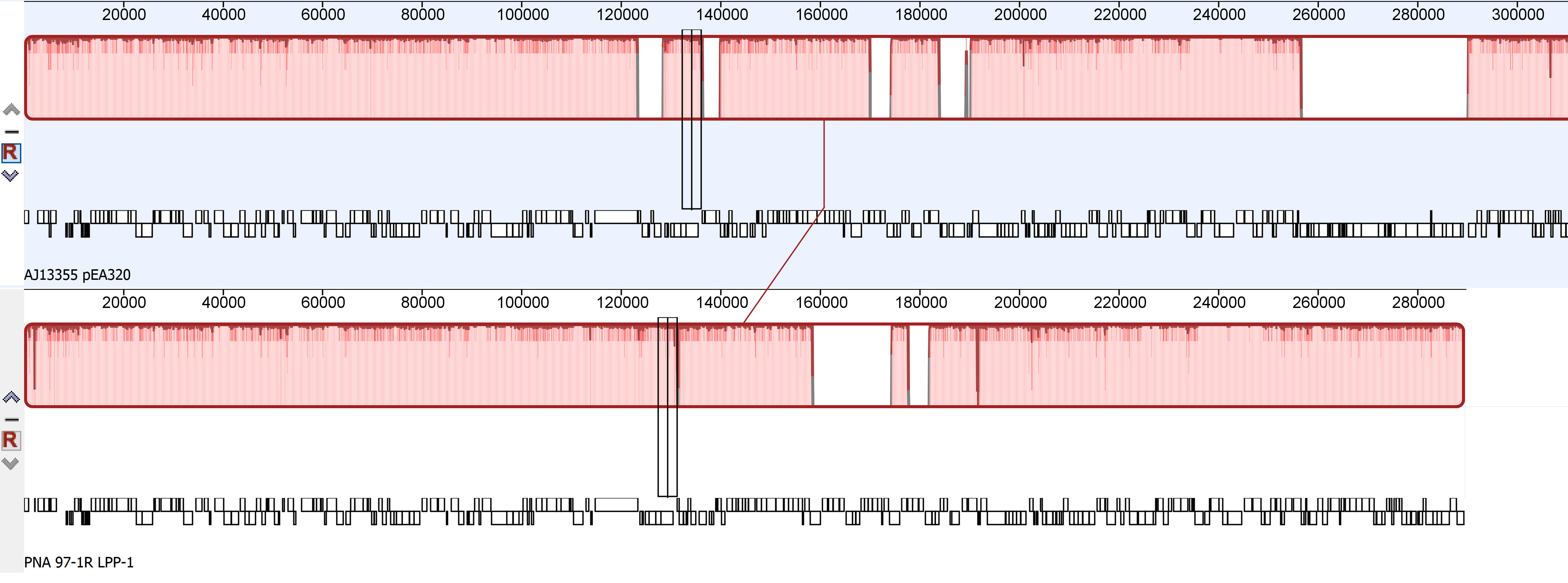

Supplement: Supplementary Figure 2 — Large Pantoea Plasmid LPP-1 alignment of proto-type strain PNA 97-1 with AJ13355 pEA320 (Accession CP020943; NC_017533.1) Alignment generated with Geneious MAUVE plugin. [file Image2.PNG]

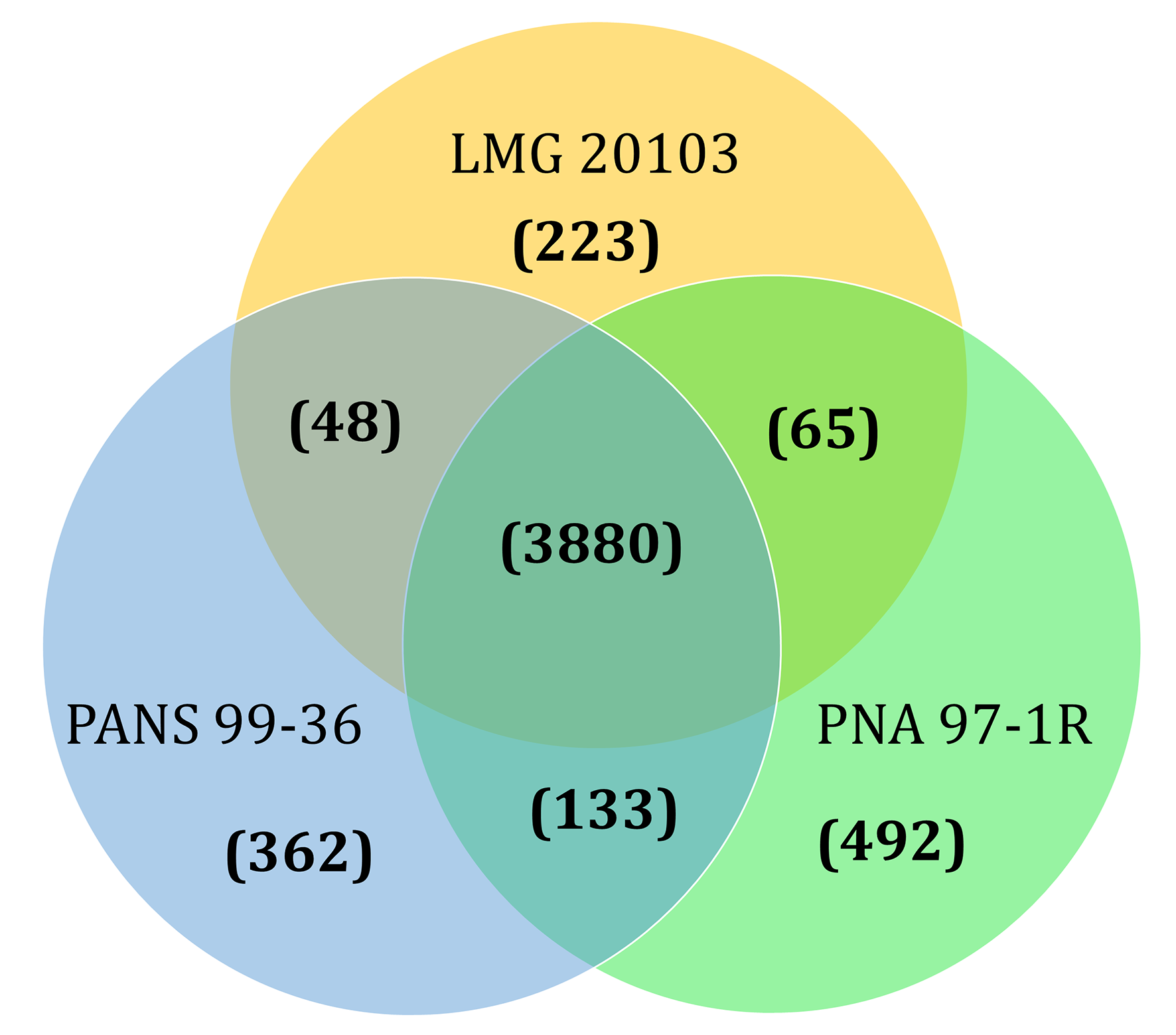

Supplement: Supplementary Figure 3 — Venn diagram of genes shared among the pathogenic isolate PNA 97-1R; nonpathogenic isolates PANS 99-36, and genomic type strain LMG 20103 (Accession CP001875.2). The number of coding sequences are derived from the gene_presence_absense.csv output of the ROARY pan-genome analysis using default parameters (Supplementary File 2). [file Image3.TIF]

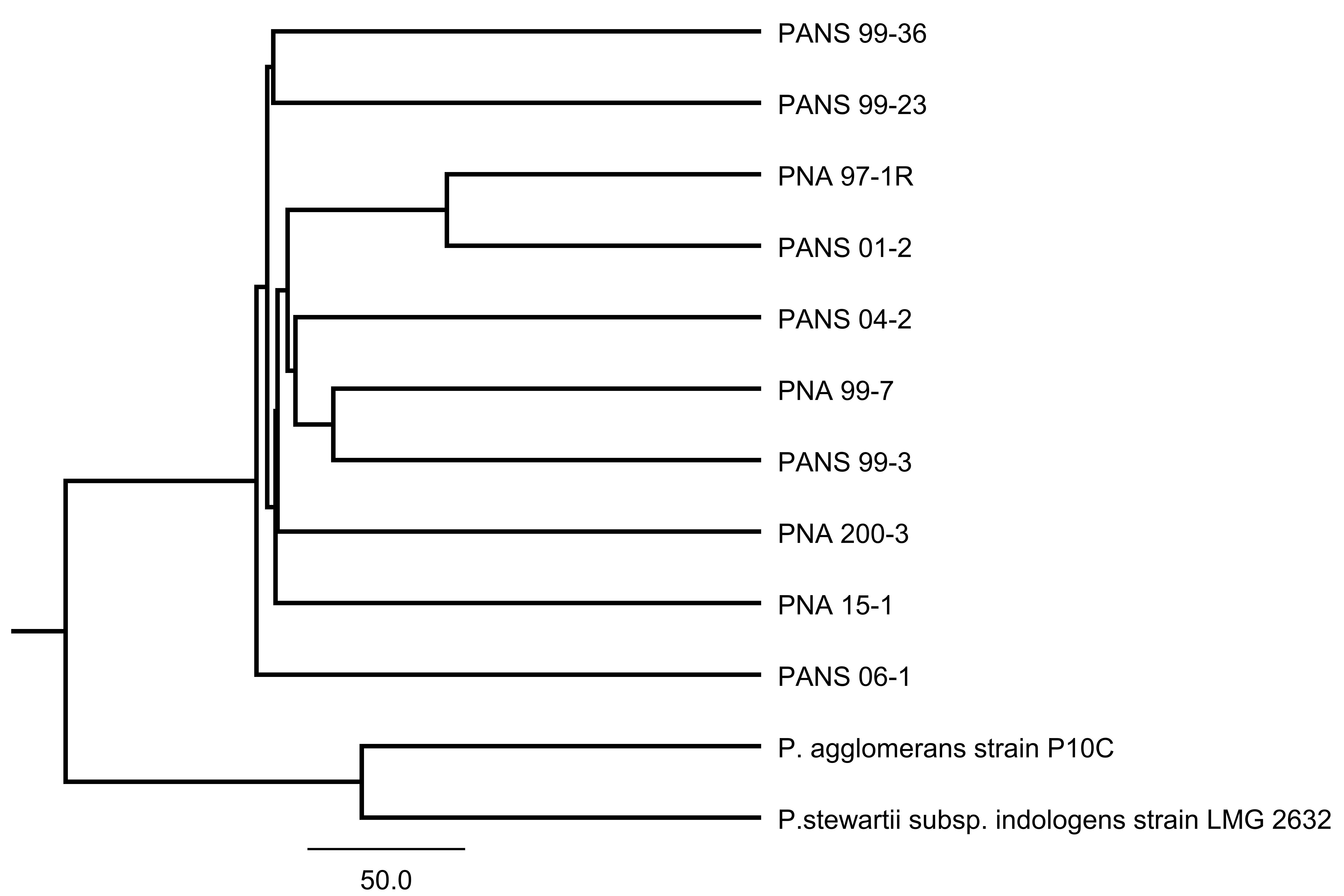

Supplement: Supplementary Figure 4 — wgMLST cladogram of Pantoea strains generated through PGAdb-builder platform. Outgroups P. agglomerans P10C and P. stewartii subsp. indologens LMG 2632 (Accession NZ_LIME00000000.1 and JPKO00000000.1). [file Image4.PNG]

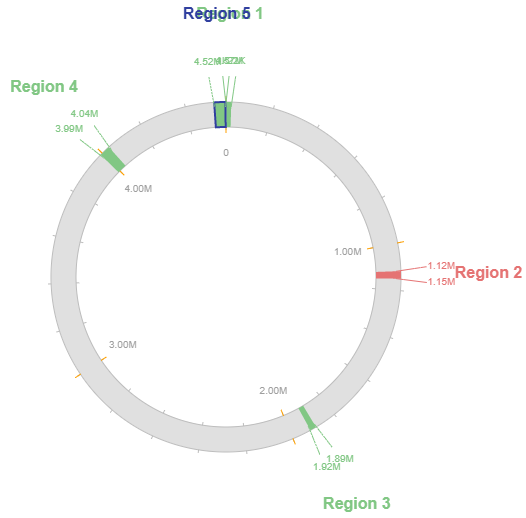

Supplement: Supplementary Figure 5 — Phaster predicted phage regions in PNA 97-1R. Green indicated intact phage regions whereas red indicates incomplete phage regions. [file Image5.PNG]

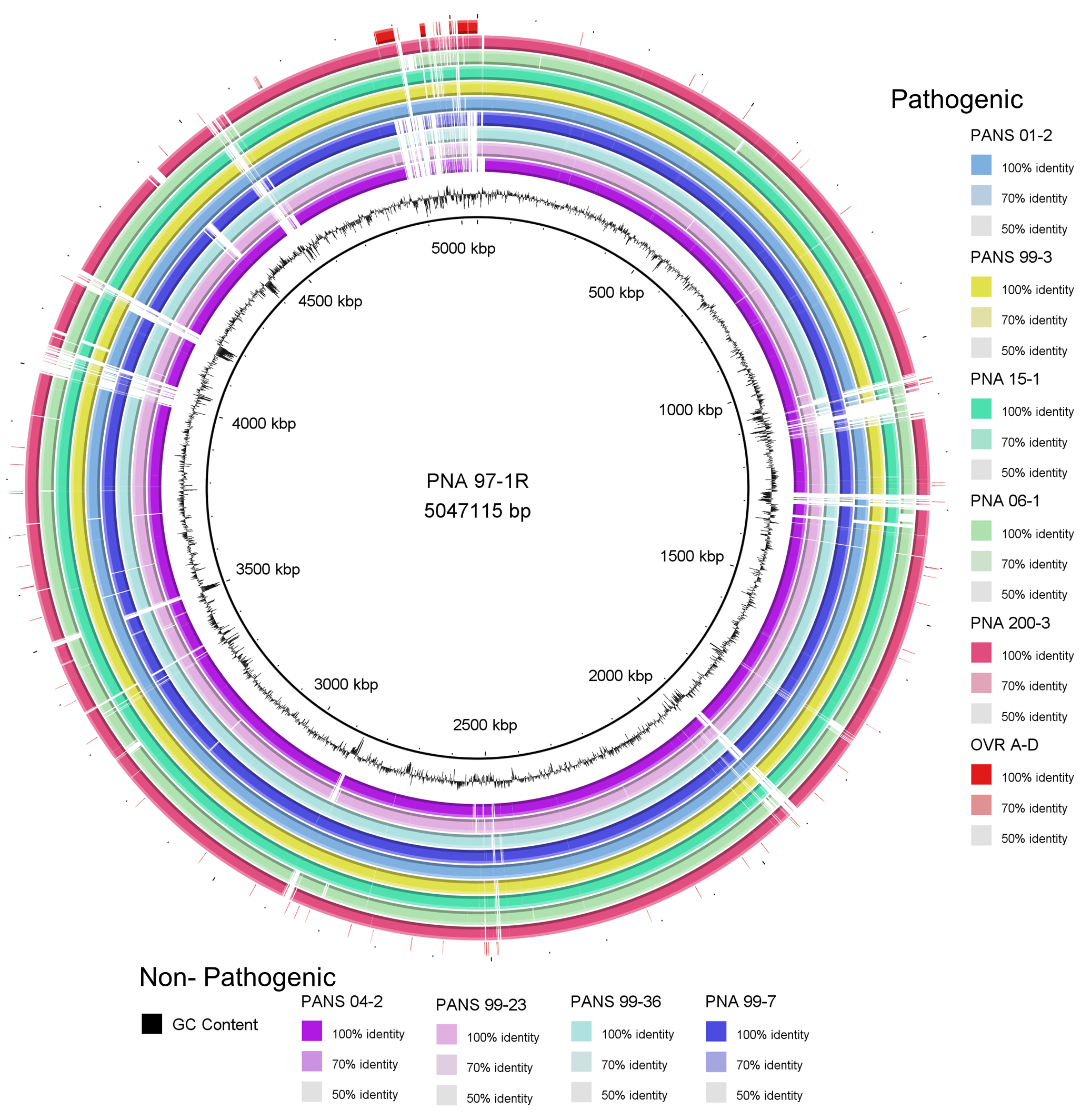

Supplement: Supplementary Figure 6 — BRIG analysis of P. ananatis strains and the OVR A-D loci to the reference strain PNA 97-1R. BRIG is a locally run analysis that generates figures based on blastn comparisons of query genomes to a reference strain. [file Image6.PNG]
