# Supplementary material for: Pantoea ananatis Genetic Diversity Analysis Reveals Limited Genomic Diversity as Well as Accessory Genes Correlated with Onion Pathogenicity
Source: Front Microbiol. 2018 Feb 13;9:184. doi: 10.3389/fmicb.2018.00184 (PMC5817063; doi:10.3389/fmicb.2018.00184)
Supplement: Supplementary Table 1 — Sequencing and assembly statistics for Illumina draft-genomes of Pantoea ananatis strains. [file Table1.DOCX]

Supplementary Table 1. Sequencing and assembly statistics for Illumina draft-genomes of *Pantoea ananatis* strains

| Species | Strain | Average Insert Size | Average Coverage | Number of reads | Number of reads w/insert size> 300 |  |  |  |  |  |  |  |
| --- | --- | --- | --- | --- | --- | --- | --- | --- | --- | --- | --- | --- |
| TRIMMED SEQUENCING STATISTICS | |  |  |  |  |  |  |  |  |  |  |  |
| *P. ananatis* | PANS 99-3 | 577 | 52.0988 | 623950 | 404911 |  |  |  |  |  |  |  |
| *P. ananatis* | PANS 99-23 | 612 | 50.9553 | 582052 | 406564 |  |  |  |  |  |  |  |
| *P. ananatis* | PANS 99-36 | 344 | 72.0018 | 878590 | 439975 |  |  |  |  |  |  |  |
| *P. ananatis* | PANS 01-2 | 615 | 73.3841 | 824793 | 587722 |  |  |  |  |  |  |  |
| *P. ananatis* | PANS 04-2 | 525 | 35.0597 | 409664 | 247948 |  |  |  |  |  |  |  |
| *P. ananatis* | PNA 97-1R | 489 | 28.9523 | 354376 | 215963 |  |  |  |  |  |  |  |
| *P. ananatis* | PNA 99-7 | 628 | 89.5981 | 973495 | 812076 |  |  |  |  |  |  |  |
| *P. ananatis* | PNA 200-3 | 421 | 167.46 | 2036787 | 1181698 |  |  |  |  |  |  |  |
| *P. ananatis* | PNA 06-1 | 664 | 53.1834 | 590978 | 456147 |  |  |  |  |  |  |  |
| *P. ananatis* | PNA 15-1 | 649 | 38.0261 | 437803 | 302005 |  |  |  |  |  |  |  |
| Species | Strain | # contigs >=0 bp | # contigs >=1000bp | Total length >=0 bp | Total length >=1000 bp | Largest contig | Total length | GC (%) | N50 | N75 | L50 | L75 |
| COMPARATIVE ASSEMBLY STATISTICS (QUAST) | | |  |  |  |  |  |  |  |  |  |  |
| *P. ananatis* | PANS 99-3 | 72 | 23 | 4973091 | 4953152 | 1557844 | 4956560 | 53.52 | 684462 | 269927 | 3 | 6 |
| *P. ananatis* | PANS 99-23 | 75 | 23 | 4981070 | 4963980 | 1583088 | 4968392 | 53.52 | 696462 | 275727 | 3 | 5 |
| *P. ananatis* | PANS 99-36 | 60 | 24 | 4975922 | 4962248 | 843014 | 4965986 | 53.43 | 410797 | 270114 | 5 | 8 |
| *P. ananatis* | PANS 01-2 | 81 | 30 | 4865308 | 4848126 | 690590 | 4851899 | 53.5 | 400581 | 338501 | 5 | 8 |
| *P. ananatis* | PANS 04-2 | 65 | 23 | 5115463 | 5101789 | 1129982 | 5105152 | 53.29 | 402837 | 212211 | 4 | 7 |
| *P. ananatis* | PNA 97-1R | 57 | 22 | 4996948 | 4985183 | 1205922 | 4986927 | 53.34 | 683537 | 342565 | 3 | 6 |
| *P. ananatis* | PNA 99-7 | 61 | 22 | 4923299 | 4910023 | 1222772 | 4913103 | 53.49 | 616357 | 345127 | 3 | 6 |
| *P. ananatis* | PNA 200-3 | 78 | 25 | 5011660 | 4990474 | 848810 | 4995978 | 53.52 | 565219 | 273902 | 4 | 7 |
| *P. ananatis* | PNA 06-1 | 72 | 33 | 4918362 | 4905152 | 1538242 | 4908261 | 53.51 | 691912 | 271490 | 3 | 6 |
| *P. ananatis* | PNA 15-1 | 65 | 27 | 4942636 | 4927811 | 915520 | 4929567 | 53.3 | 433957 | 223286 | 4 | 8 |
