# Supplementary material for: Pantoea ananatis Genetic Diversity Analysis Reveals Limited Genomic Diversity as Well as Accessory Genes Correlated with Onion Pathogenicity
Source: Front Microbiol. 2018 Feb 13;9:184. doi: 10.3389/fmicb.2018.00184 (PMC5817063; doi:10.3389/fmicb.2018.00184)
Supplement: Supplementary Table 2 — RAST functional categories for predicted protein coding sequences of Pantoea ananatis strains. [file Table2.DOCX]

Supplementary Table 2. RAST Functional categories for predicted protein coding sequences of *Pantoea ananatis* strains.

| CDS Function Category | PANS 99-3 | PANS 99-23 | PANS 99-36 | PANS 01-2 | PANS 04-2 | PNA 97-1^Rf^ | PNA 99-7 | PNA 200-3 | PNA 06-1 | PNA 15-1 |
| --- | --- | --- | --- | --- | --- | --- | --- | --- | --- | --- |
| Number of Coding Sequences | 4992 | 4742 | 4681 | 4720 | 4722 | 4897 | 4810 | 4862 | 4836 | 4833 |
| Number of RNAs | 106 | 87 | 96 | 96 | 100 | 100 | 95 | 96 | 105 | 94 |
| Number of Hypothetical Proteins | 1,330 | 1,165 | 1,095 | 1,138 | 1,178 | 1,266 | 1,254 | 1,244 | 1,220 | 1,259 |
| Cofactors, Vitamins, Prosthetic Groups, Pigments | 257 | 247 | 260 | 252 | 260 | 253 | 254 | 251 | 250 | 257 |
| Cell Wall and Capsule | 211 | 218 | 222 | 217 | 222 | 220 | 229 | 229 | 212 | 213 |
| Virulence, Disease and Defense | 92 | 91 | 93 | 91 | 92 | 91 | 91 | 95 | 96 | 92 |
| Potassium metabolism | 24 | 24 | 24 | 24 | 24 | 24 | 24 | 24 | 24 | 24 |
| Phages, Prophages, Transposable elements, Plasmids | 28 | 22 | 26 | 53 | 3 | 49 | 22 | 42 | 31 | 26 |
| Membrane Transport | 163 | 146 | 153 | 143 | 151 | 148 | 147 | 142 | 173 | 150 |
| Iron acquisition and metabolism | 31 | 31 | 31 | 31 | 31 | 31 | 31 | 31 | 31 | 32 |
| RNA metabolism | 213 | 213 | 211 | 215 | 210 | 211 | 211 | 212 | 213 | 213 |
| Nucleosides and Nucleotides | 131 | 129 | 128 | 129 | 128 | 130 | 128 | 129 | 130 | 130 |
| Protein Metabolism | 275 | 269 | 271 | 270 | 268 | 273 | 272 | 272 | 270 | 269 |
| Cell Division and Cell Cycle | 41 | 43 | 44 | 41 | 44 | 43 | 44 | 46 | 42 | 44 |
| Motitlity and Chemotaxis | 133 | 131 | 130 | 132 | 131 | 136 | 135 | 134 | 140 | 133 |
| Regulation and Cell signaling | 133 | 133 | 138 | 135 | 136 | 135 | 137 | 142 | 134 | 135 |
| Secondary Metabolism | 5 | 5 | 5 | 5 | 5 | 5 | 5 | 5 | 5 | 5 |
| DNA Metabolism | 114 | 158 | 111 | 115 | 118 | 123 | 127 | 119 | 155 | 110 |
| Fatty Acids, Lipids, and Isoprenoids | 175 | 170 | 172 | 161 | 169 | 164 | 160 | 160 | 138 | 170 |
| Nitrogen Metabolism | 21 | 21 | 21 | 21 | 21 | 21 | 20 | 21 | 21 | 21 |
| Dormancy and Sporulation | 5 | 5 | 5 | 5 | 5 | 5 | 5 | 5 | 5 | 5 |
| Respiration | 110 | 108 | 108 | 110 | 108 | 110 | 109 | 109 | 111 | 109 |
| Stress Response | 165 | 164 | 161 | 165 | 163 | 165 | 162 | 163 | 168 | 165 |
| Metabolism of Aromatic Compounds | 26 | 20 | 38 | 26 | 20 | 26 | 22 | 24 | 24 | 24 |
| Amino Acids and Derivatives | 461 | 455 | 446 | 453 | 455 | 462 | 449 | 458 | 465 | 454 |
| Sulfur Metabolism | 67 | 65 | 65 | 65 | 66 | 66 | 66 | 67 | 66 | 65 |
| Phosphorus Metabolism | 62 | 62 | 61 | 60 | 64 | 60 | 59 | 61 | 59 | 61 |
| Carbohydrates | 633 | 604 | 619 | 619 | 606 | 636 | 603 | 633 | 612 | 622 |
