# Supplementary material for: Pantoea ananatis Genetic Diversity Analysis Reveals Limited Genomic Diversity as Well as Accessory Genes Correlated with Onion Pathogenicity
Source: Front Microbiol. 2018 Feb 13;9:184. doi: 10.3389/fmicb.2018.00184 (PMC5817063; doi:10.3389/fmicb.2018.00184)
Supplement: Supplementary Table 3 — Primers and conditions used for amplification and sequencing. [file Table3.docx]

Supplementary Table 3. Primers and conditions used for amplification and sequencing

| Gene | Primer  name | Primer sequence | Primer positions on coding sequences | PCR cycles | Template size (bp)^a^ | Template position on *P. ananatis* PA13 genome^b^ |
| --- | --- | --- | --- | --- | --- | --- |
| *fusA* | fusA3 | 5’-CAT CGG TAT CAG TGC KCA CAT CGA-3’ | 36-59 | 2 min 94°C; 1 min 94°C, 1 min 58°C, 1 min 72°C (31 cycles); 5 min 72°C | 639 | 4449773-450416 |
|  | fusA4 | 5’-CAG CAT CGC CTG AAC RCC TTT GTT-3’ |  |  |  |  |
| *gyrB* | gyrB3 | 5’-GCG TAA GCG CCC GGG TAT GTA-3’ | 57-77 | 2 min 94°C; 1 min 94°C, 1 min 58°C, 1 min 72°C (31 cycles); 5 min 72°C | 427 | 4108-4543 |
|  | gyrB4 | 5’CCG TCG ACG TCC GCA TCG GTC AT-3’ | 1488-1508 |  |  |  |
|  | gyrB3i | 5’-AAC GCW ATC GAC GAA GC-3’ | 136-152 | Primers used only for sequencing |  |  |
|  | gyrB4i | 5’-TGG AAC CCR TCR TTC CAC-3’ | 771-788 |  |  |  |
| *leuS* | leuS3 | 5’-CAG ACC GTG CTG GCC AAC GAR CAR GT-3’ | 487-512 | 2 min 94°C; 1 min 94°C, 1 min 58°C, 1 min 72°C (31 cycles); 5 min 72°C | 640 | 3282463-3283102 |
|  | leuS4 | 5’-CGG CGC GCC CCA RTA RCG CT-3’ | 1274-1293 |  |  |  |
| *pyrG* | pyrG3 | 5’-GGG GTC GTA TCC TCT CTG GGT AAA GG-3’ | 31-56 | 2 min 94°C; 1 min 94°C, 1 min 58°C, 1 min 72°C (31 cycles); 5 min 72°C | 316 | 1019655-1019970 |
|  | pyrG4 | 5’-GGA ACG GCA GGG ATT CGA TAT CNC CKA-3’ | 434-460 |  |  |  |
| *rplB* | rplB3 | 5’-CAG TTG TTG AAC GTC TTG AGT ACG ATC C-3’ | 227-254 | 2 min 94°C; 1 min 94°C, 1 min 58°C, 1 min 72°C (31 cycles); 5 min 72°C | 343 | 457307-457649 |
|  | rplB4 | 5’-CAC CAC CAC CAT GYG GGT GRT C-3’ | 685-706 |  |  |  |
| *rpoB* | Vic3 | 5’-GGC GAA ATG GCW GAG AAC CA-3’ | 1422-1442 | 4 min 94°C; 30 sec 94°C, 30 sec 50°C, 30 sec 72°C (31 cycles); 5 min 72°C | 596 | 4432827-4433422 |
|  | Vic2 | 5’-GAG TCT TCG AAG TTG TAA CC-3’ | 2469-2489 |  |  |  |

^a^Size of the sequence template of the internal portion of PCR product used for sequence comparison.

^b^ Location of each gene sequence analysis on *P. ananatis* PA 13 reference genome (NCBI genome accession no. CP003085).
