# Supplementary material for: Pantoea ananatis Genetic Diversity Analysis Reveals Limited Genomic Diversity as Well as Accessory Genes Correlated with Onion Pathogenicity
Source: Front Microbiol. 2018 Feb 13;9:184. doi: 10.3389/fmicb.2018.00184 (PMC5817063; doi:10.3389/fmicb.2018.00184)
Supplement: Supplementary Table 4 — IslandPath DIMOB genomic islands identified in WGS P. ananatis strains. [file Table4.DOCX]

Supplementary Table 4. IslandPath DIMOB genomic islands identified in WGS *P. ananatis* strains

| Strain | Pathogenicity | Predicted Genomic Islands | | Relative Size of Genomic Islands (bp) |  |
| --- | --- | --- | --- | --- | --- |
| PANS 99-3 | + | 13 | 9,060-97,342 | | |
| PANS 99-23 | - | 9 | 6,804-89,159 | | |
| PANS 99-36 | - | 9 | 5,430-144,177 | | |
| PANS 01-2 | + | 10 | 6,804-101,463 | | |
| PANS 04-2 | - | 9 | 10,043-133,083 | | |
| PNA 97-1R | + | 9 | 6,961-115,320 | | |
| PNA 99-7 | - | 10 | 6,804-101,463 | | |
| PNA 200-3 | + | 15 | 6,075-64,868 | | |
| PNA 06-1 | + | 13 | 6,498-98,967 | | |
| PNA 15-1 | + | 13 | 6,085-150,125 | | |
